# Supplementary material for: Multiple timescales of temporal context in risky choice: Behavioral identification and relationships to physiological arousal
Source: PLoS One. 2024 Jan 19;19(1):e0296681. doi: 10.1371/journal.pone.0296681 (PMC10798524; doi:10.1371/journal.pone.0296681)
Supplement: S1 Text — (DOCX) [file pone.0296681.s001.docx]

**Supporting Information for “Multiple timescales of temporal context in risky choice: Behavioral identification and relationships to physiological arousal”**

Hayley R. Brooks^1,2^ & Peter Sokol-Hessner^1^*

^1^Department of Psychology, University of Denver, Denver, Colorado, United States of America

^2^Department of Cognitive, Linguistic, and Psychological Sciences, Brown University, Providence, Rhode Island, United States of America

*Corresponding author

Email: ﻿Peter.Sokol-Hessner@du.edu (PSH)

**Effect of outcomes on subsequent risk-taking**

While recent outcomes may play a fundamental role in risk-taking, the direction of the outcome effect is variable across studies with large previous gains leading to decreases [1–3] or a combination of decreases and increases [4–7] in risk-taking on the subsequent choice. Understanding the factors contributing to this variability is complicated by the fact that most studies with trial-by-trial feedback do not address the effects of outcomes on subsequent risk-taking [8–21] but see [22].This variability may be related to the effects of contextual sensitivity, among other factors (e.g. how the outcome is realized [22]), but a thorough review is beyond the scope of the current article.

**Additional citations for studies that actively design against global timescale effects**

It was noted in the main text that many studies pay the outcome of a single trial or subset of trials. Due to space limitations, we included representative citations in the main text. Here, we provide a more extensive (but not fully exhaustive) list of references to studies that actively design against global timescale effects by paying for singular or small subsets of the trials completed in the task ([2,4,8,10,11,20,23–38]. All but two of these studies do not analyze and report the influence of cumulative earnings on risky decision-making [2,4,8,10,11,20,23–31,33–37]. Because so few studies have even examined the effects of recent events on subsequent risks, it is possible that contextual effects have been present in many datasets, but were simply not analyzed.

**Task length variation**

We increased the length of our risky decision-making task twice in the beginning of data collection to increase the quantity of data collected per-participant. These changes meant we were able to incorporate additional trials and context shifts into the task. As a result, the first 8 participants completed 171 trials (for a total of 9 runs), participants 9-13 (N = 5) completed 204 trials (for a total of 10 runs) and participants 14-62 (N = 49) completed 240 trials (for a total of 10 runs). No changes were made to the structure, content, or timing of the task, beyond increasing the number of trials completed by each participant.

**Shift size**

The nine possible absolute shift sizes (in EV) in between each run were $5, $6.25, $7.50, $8.75, $10, $11.25, $12.50, $13.75, $15. For each participant, the order of shift sizes was randomized, and each shift size was either positive or negative depending on the EV level of the previous run to ensure values of choice options did not go below $0 or above +$70. The first 15 trials in our choice set gradually increased (in EV) from $.50 up to $30 in increments of $2 to consistently expose all participants to the wide range of values in our choice set before they underwent their unique pseudorandom set of runs and shifts.

**Immediate timescale in risk-taking: Past outcome multiple trials back**

In a previous analysis (Brooks & Sokol-Hessner, 2020, *Scientific Reports*), the effect of past outcome amount on risk-taking was found to be brief, extending only to the previous trial. In the current analysis, we tested whether the risk-taking changed as a function of past outcome amounts from one, two, and three trials back using a linear mixed effects model (choice ~ 0 + outcome_t-1_ + outcome_t-2_ + outcome_t-3_ + (1 | subject)). We replicated previous research (Brooks & Sokol-Hessner, 2020), showing that the effect of past outcome amount on risk-taking is short-lasting (outcome_t-1_ *β* = -.3(.09), p = .0004_;_ outcome_t-2_ *β* = .15(.08), p =.09)_;_ outcome_t-3_ *β* = .09 (.08), p =.28; Full regression results are below in SI Table 2). Taken together, these independent and convergent findings strongly suggest that the past outcome effect is indeed brief.

**Relationship between outcome amount and positive shift**

In the main text, we report a relative increase in risk-taking following 1) small previous outcome amounts (relative to large previous outcome amounts) and 2) large positive shifts (relative to no shifts). The intentional temporal structure of the choice set means that the range of past outcome amounts associated with large positive shifts is smaller than the range of past outcomes associated with small positive shifts. This design feature could potentially introduce a confound where the increase in risk-taking following a positive shift is not due to the change in value between runs but is accounted for by the effect of past outcome (more risk-taking following small outcomes). In other words, following a large positive shift, the previous outcome is slightly more likely to be relatively smaller than outcomes following small positive shift, which could therefore lead to more risk-taking. We tested this possibility quantitatively with a step-wise regression, starting from model 2 reported in the main text (which was the 2^nd^ step in a conservative two-step procedure, regressing previous outcome amount on choice, holding estimates for current-trial variables constant). We then followed the same conservative two-step approach described in the results section of the main text a third time to calculate the predicted values from the past outcome model (model 2). We then incorporated those predicted values in a third regression in which we regressed choice on positive shift amount, using the offset function in R to include the predicted values from the past outcome model (model 2). This approach gave all of the shared variance between previous outcomes and positive shifts to the previous outcome variable. If the effect of positive shift could be entirely accounted for by the past outcome effect, then we would expect to see no significant effect of shift on risk-taking. However, we replicated the positive shift effect (positive shift amount_t_ *β* = 4.72(.99), *p* =1.83x10^-6^), indicating that increases in risk-taking following large positive shifts are distinct from the effect of previous outcomes (full regression results are in SI Table 2).

We also tested for an interaction between past outcome and positive shift amount and found only the main effects for outcome and positive shift as reported in the main text (outcome_t-1_ *β* = -0.17 (.06), p = .005; positive shift_t_ = 6.21(1.43), *p* = 1.48x10^-5^; outcome_t-1_ x positive shift_t_ *β* = -7.71(5.46), p =.16). We report the full regression results in SI Table 2. Together, this demonstrates that the effects of outcome amount and positive shift on risk-taking are additive but distinct.

**Testing an alternative trial-level model: Expected value difference**

In the current trial-level variables model (model 1) reported in the main text, we regressed risky choice on risky gain($), safe($), and magnitude($). We then held those regression estimates constant as we estimated the effects of temporal context on risk in subsequent models. That risky gain, safe, and magnitude change together with the value of a given run across the task introduced collinearity into this initial regression.

Collinearity can of course lead to issues interpreting parameter estimates for a given regressor that is collinear with others. Critically, for the purposes of the present research, the parameter estimates in question (risky gain, safe, and magnitude) both showed expected effects (e.g., effects for which we have very strong priors like observing more risk-taking as risky gain amounts rise), and perhaps most importantly, were not the central focus of our analysis. Our goal was to account for these factors, and then assess the additional impact of contextual variables. It is additionally noteworthy that the literature on how to address collinearity is equivocal as to whether any of a variety of possible corrective approaches are actually helpful, or if they just introduce additional issues [39,40].We interpret this literature to persuasively argue that when collinearity occurs, it is critical to explicitly acknowledge and discuss it as a limitation, but that there is ultimately no agreed-upon way to eliminate collinearity without introducing other issues.

To test whether the temporal context effects in risky decision-making were significantly impacted by collinearity of the current trial-level model, we eliminated the collinearity by replacing model 1 with an alternative model that only included the expected value difference (mean expected value of the gamble – mean expected value of the safe option). As expected, we found that risk-taking robustly increased as the expected value difference increased (*β* = 32.04 (2.913), p < 2x10^-16^; full regression results are shown in SI Table 2). We followed the same conservative two-step approach as described in detail in the main text and held constant the parameter estimate for expected value difference. In a subsequent model, we regressed choice on past outcome amount_t-1_, positive shift amount_t_, and earnings_t_ relative to expectations_t_ (which constituted one of the final models in the main text) and tested whether the effect of these variables on risk-taking were qualitatively different from the effects we reported in the main text. We replicated the effects reported in the main text: risk-taking increased as past outcome decreased (*β* = -.42 (.09), p = 9.7x10^-7^), positive shift increased (*β* = 4.37 (.99), p = 1.07x10^-5^), and when earnings were more than expected (earnings *β* = 1.39 (.43), p =.001, expectations *β* = -.77 (.33), p =.02; Full regression results in SI Table 2). Here we demonstrate that any collinearity in the trial-level model (risky gain, safe, and magnitude) does not impact the main findings that risky decision-making is contextually sensitive at multiple timescales.

**Global timescale in risk-taking: Earnings relative to expectations**

As reported in the Results section of the manuscript, we examined how risk-taking changed as a function of the relationship between earnings relative to linear expectations (Model 4a in the main text and model 13 below in the complementary reanalysis). In our models, we include a term for both earnings and expectations. This approach examines, rather than assumes, the relative weighting of earnings and expectations and highlights how changes in risk-taking for a given earning state depends on expectations (see SI Figure 1 for a schematic example). If earnings exceed expectations (green line in SI Fig. 1), participants become more likely to take risks as the gap increases but if the same level of earnings falls behind expectations (red line), participants instead become less likely to take risks. Our approach demonstrates how the relative weight of earnings and expectations can produce different behavioral outcomes, which would not be possible with a single regressor that combines earnings and expectations and as a consequence assumes their relative weighting.

**SI Fig. 1. Risk-taking depends on earnings relative to expectations.** Each line shows a different relative weighting between earnings and expectations in a hypothetical scenario consistent with observations in the study. When earnings outpace expectations (green line), participants become more likely to take risks, whereas when the same level of earnings falls below expectations (red line), participants become less likely to take risks at the same earnings amount (vertical dotted line). These lines are simulated based on hypothetical earnings and linear expectations. Modeling both expectations and earnings (versus combining expectations and earnings into a single regressor) avoids the assumption about their relative weighting and allows this unique pattern of the effects of earnings relative to expectations on risk-taking.

**Global timescale in risk-taking: Piecewise linear expectations**

In the Results section of the manuscript, we examined the effect on risk-taking behavior of cumulative earnings relative to expectations and estimated the slope of an assumed linear expectation term for simplicity. It is possible (and indeed likely) that in some settings, expectations do not change in a purely linear fashion. For example, in the current study, choices were distributed around systematically-varied “EV levels”, and it is possible that expectations increased proportional to the current EV level. This would imply piecewise linear expectations. To examine this possibility empirically, we conducted an additional analysis that examined whether cumulative earnings, relative to a piecewise linear expectation term, better accounted for risk-taking behavior compared to a purely linear expectation term. The piecewise linear expectation term is best understood as adjusting to the level of context (i.e. EV = $5, $10, $15, $20, $25) in the task. For example, expectations would increase more steeply across trials when the EV level was $25 relative to when it was $5.

We conducted an identical analysis as Model 4a regressing on choice_t_ four variables: outcome_t-1_, positive shift_t_, cumulative earnings_t_, and piecewise linear expectation_t_ (substituting the linear expectation_t_ variable). While we found no main effect of cumulative earnings (*β* = 1.09(.683), *p* = .11) or the piecewise linear expectation (*β* = -.75(.69), *p* = .28), the main effects of outcome (*β* = -.25(.09), *p* =.005) and positive shift (*β* = 5.22(1.04), *p* = 5.3 x 10^-7^) remained (AIC = 13990.0). The AIC of this model was a slight improvement from model 4a (which had an AIC = 13991.1) suggesting that expectations may have increased in this piecewise linear manner. However, we urge caution in interpreting these results, as the linear regression framework is not ideal for estimating possible non-linear expectation effects. We hope that future studies can more directly examine the possibility of globally nonlinear (whether locally linear or not) changes in expectations with appropriate nonlinear models fit with maximum likelihood estimation or Hierarchical Bayesian techniques.

**Skin conductance responses for all participants (responders and non-responders)**

In the Results section of the manuscript, we tested the relationship between SCRs to outcomes, temporal context and risk-taking in responders only (N=46 with a total 10,304 trials of which 5,017 were “responding” trials with SCRs greater than zero). Here we tested whether conducting an identical analysis with all participants qualitatively changed our findings (N=61 with a total of 13,840 trials of which 5,507 were “responding” trials). Model numbers correspond to the model numbers in the Results section of the manuscript (e.g. the version of “model 5b” in the manuscript that here includes all participants is “model 5b full” in this section).

Note that we think these results should be interpreted with caution. It is unclear whether the inclusion of “non-responder” participants adds meaningful data (e.g. SCR of 0$\mu$S means that a participant did not respond to earnings) or that the lack of consistent responses reflects other factors not directly related to the study (e.g. nonspecific determinants of skin conductance like the participant’s body temperature relative to the ambient air, or global arousal levels).

Similar to the results for responders only, participants demonstrated a range of mean SCRs following wins (.01-.38$\mu$S), losses (0-.33$\mu$S), and safe (.01-.34 $\mu$S) outcomes. Three paired-sample t-tests revealed no significant difference in mean SCRs following wins (M = .14, SD = .09), losses (M = .14, SD = .09), and safe (M=.13, SD = .07) outcomes (wins v. losses: *t*(60)=-.21, p=.84; wins v. safe: *t*(60)=.8, *p*=.43; losses v. safe: *t*(60) = .97, *p* = .33).

In the analysis with responders only, we tested whether SCRs to outcomes were related to three timescales of temporal context. While we noted no significant relationship between SCR_t_ and outcome_t_ (model 5a) or SCR_t_ and positive shift_t_ (model 5b), there was a positive significant effect of cumulative earnings_t_ on SCR_t_ (model 5c). Testing models 5a-c in the dataset with both responders and non-responders revealed slightly different results. At the immediate timescale, we noted a significant, negative effect of outcome_t_ on outcome SCR_t_ (model 5a full; outcome_t_ *β* = -.017(.0075), p=.02). This result suggests that SCRs increased following smaller outcomes, however, it is unclear whether such effect occurred due to a floor effect given the gain-only nature of the task (e.g. SCRs larger for loss relative to gain outcomes), the addition of non-responding trials (e.g. SCR = 0$\mu$S for a large outcome), or some other factor. At the neighborhood timescale, we found no significant relationship between SCR_t_ and positive shift_t_ (model 5b full; positive shift_t_ *β* = -.08(.08), *p* = .33). At the global timescale, we identified a positive effect of cumulative earnings (earnings_t_) on SCR_t_ (model 5c full: earnings *β* = .11(.008), *p* < 2 x 10^-16^ AIC = -4536.2) but the effect is not significant when accounting for linear expectation_t_ (earnings_t_ *β* = .05(.04), *p*=.2; trial_t_ *β* = .05(.03), *p* = .06; AIC = -4537.6). We also examined whether cumulative earnings_t_ and outcome_t_ interacted to influence SCR_t_ (model 5d full) and while the main effect of earnings_t_ remained (*β* = .11(.01), *p* < 2 x 10^-16^), we found no significant interaction between earnings_t_ and outcome_t_ (*β* = .005(.03), *p* = .87).

We next tested whether physiological arousal responses to outcomes accounted for risk-taking behavior in addition to the three timescales of temporal context. In the responders-only dataset, we found no additional main effect of SCRs_t-1_ (model 6a) and no interaction between SCRs_t-1_ and outcome_t-1_ (model 6b) on choice_t_ in addition to the three timescales of temporal context. Adding data from non-responders did not change these results (model 6a full: SCR_t-t_ *β* = -.06(.1), *p* = .5; model 6b full: SCR_t-t_ x outcome_t-1_ *β* = -.15(.38), *p* = .7).

The relationship between SCRs to outcomes, temporal context, and risk-taking varied slightly when adding SCRs from non-responders. We found that the relationship between outcome_t-1_ and SCR_t_ was stronger, the relationship between SCR_t_ and positive shift_t_  did not vary, and the relationship between cumulative earnings_t_ and SCR_t_ was weaker when accounting for SCRs from non-responders.

**Skin conductance responses during the decision phase including all non-responders**

In the Results section of the manuscript, we conducted an exploratory analysis on the relationship between SCRs during the decision phase, temporal context and risk-taking in responders only (N=26 with a total 5,832 trials of which 2,367 were “responding” trials with SCRs greater than zero). Here we tested whether an identical analysis with all participants qualitatively changed our findings (N=61 with a total of 13,840 trials of which 3,499 were “responding” trials). While all data are present in this analysis, it should still be considered exploratory as SCRs during the decision phase are difficult to interpret, relative to SCRs following outcomes as participants processed and decided between two choice options during a relatively short window of time. Model numbers correspond to those in reported in the manuscript.

Participants demonstrated a range of mean SCRs when taking risks (0-.36$\mu$S) and when rejecting risks for the guaranteed alternative (0-.33 $\mu$S). Similar to the analysis of responders-only, we found no overall significant difference in mean SCRs by decision type (paired-samples Wilcoxon signed-rank test: gamble M = .09, SD = .07; guaranteed alternative M = .09, SD = .07; *V*=932, *p*=.9) and no significant change in SCRs as a function of risk-taking (choice *β*= .0004(.002), *p*=.81; Model 7 full). Adding non-responders’ SCRs during the decision phase to the analysis did not change the finding that physiological arousal responses were not directly related to accepting or rejecting the gamble.

In the manuscript, we tested whether SCRs during the decision-phase were related to three timescales of temporal context. We noted that SCRs did not change as a function of outcome or cumulative earnings but increased following positive shifts (model 8a). Accounting for non-responders did not qualitatively change the results (model 8a full). We identified no effect of outcome_t-1_ (*β*= -.007(.007), *p* = .3), a main effect of positive shift_t_ (*β* = .17(.07), *p* = .02), and no effect of cumulative earnings_t_ (*β* = -.02(.03), *p* = .55) or linear expectation_t_ (*β* = .02(.03), *p* = .45) on SCR_t_. Across both analyses, SCRs during the decision phase varied at the neighborhood level of context but not the immediate or global levels of temporal context.

In the responders-only analysis, the effect of positive shift was short-lasting (model 8b) and did not interact with choice (model 8d). These results did not change when accounting for non-responders. The positive shift effect on SCRs during the decision phase was short-lasting, dropping off after the trial following the shift (model 8b full: positive shift_t_ *β*= .15(.07), *p*=.047; positive shift_t-1_ *β*= -.11(.07), *p*=.13; positive shift_t-2_ *β*=.03(.07), *p*=.67; positive shift_t-3_ *β*= -.06(.07), *p*=.4; positive shift_t-4_ *β*= -.05(.07), *p*=.49; positive shift_t-5_ *β*= -.14(.07), *p*=.07; Figure 5b in main text). Similar to the responders-only analysis, we identified no interaction between positive shift and choice (model4c full; positive shift_t_ x choice_t_ *β*= .13(.07), *p*=.09).

Finally, we tested whether accounting for non-responders changed the results that SCRs during decision-phase did not account for risk-taking behavior in addition to the three levels of temporal context previously identified (model 8c). When accounting for non-responders, the main effects of outcome_t-1_ (*β*= -.7(.11), *p*=4.8 x 10^-10^), positive shift_t_ (*β*= 5.2(1), *p*=.0000004), and the interaction between outcome_t-1_ and earnings_t_ (*β*= 1.3(.29), *p*=.00001) remained significant but we identified no significant effect of SCRs_t_ (*β*= -.03(.1), *p*=.8) on risk-taking nor main effect of cumulative earnings (*β*= .06(.07), *p*=.44).

Including skin conductance responses during the decision-phase from both responders and non-responders did not qualitatively change the relationship between SCRs during the decision-phase, temporal context and risk-taking.

## **Complementary re-analysis**

### **Data**

To complement the data collection in the main text focused on physiological arousal responses, we re-analyzed binary choice data from a double-blind, placebo-controlled, within-subjects study examining how the administration of the beta-adrenergic receptor antagonist propranolol, which affects the neurohormonal basis of arousal responses [9], changed decision-making. In the study, 47 participants (22 females; mean age = 26.6(5.1), median age = 25, range: 19-38) recruited from the New York City area participated in two decision-making sessions. Participants received a pill containing a dose of propranolol (80mg) in one session and a placebo pill in the other, order pseudorandomly counterbalanced across participants. In both sessions, participants completed a risky monetary task. After removing missed trials (a total of 66 trials were missed across 31 out of 47 participants with the median participant missing 1 trial), the dataset comprised a total of 14,026 choices.

### **Task**

The risky monetary decision-making task completed on each day consisted of 150 trials offering a choice between a risky gamble (possible gain and loss outcomes with equal probability) and a safe option. Each trial was one of two types: gain-only trials (30/150 trials) consisted of a risky gamble yielding a possible gain value up to $30 and a loss of $0 and a safe amount up to $12, and mixed valence trials (120/150 trials) consisted of a risky gamble yielding a possible gain amount ranging from $2 to $12 and a possible loss amount ranging from -$.50 to -$24 and a safe amount of $0. On each trial, participants viewed the choice options for 2s, followed by a response window of 2s. Once participants entered their decision, there was an interstimulus interval (1s) after which the outcome was displayed for 1s, followed by a variable intertrial interval between 1s and 3s (See the Supplementary Materials Table 1 for task similarities and differences in main text and this reanalysis). Data from this paper can be accessed at: <https://osf.io/a7nvx/> [41]. For all data cleaning and analysis scripts: <https://github.com/sokolhessnerlab/vic> [42].

**Results**

Identical to the analysis in the main text, we fit generalized linear binomial mixed effects models to the binary risky choice data to examine the effects of temporal context on risk-taking and whether these effects changed as a function of the pharmacological manipulation of physiological arousal.

The risky decision-making task was very similar to that used in the main text, with participants choosing on each of many trials between a risky gamble with two possible outcomes received with equal probability and a guaranteed or safe alternative. As in the main text, participants also received trial-by-trial feedback and a payout based on the outcomes from a single trial or a subset of trials. The task in Sokol-Hessner et al (2015) did include both gain and loss possible outcomes and lacked intentional temporal structure.

The lack of intentional temporal structure affected our analyses of the Sokol-Hessner et al (2015) data in two ways. First, the conservative two-step approach used in the main text was not necessary given that the values encountered on a given trial were not highly correlated with those on previous or following trials. Second, at the neighborhood timescale, given the task contained neither runs of similar trials, nor shifts between runs, we were only able to analyze such ‘runs’ as might occur by chance in the random ordering of trials. To do this, we conducted an exploratory analysis testing for potential changes in risk-taking as a function of the difference between the mean EV on the current trial and 1) the mean EV on the previous trial, or 2) the mean EV averaged across several previous trials (i.e. 3 trials; 20 trials).

Data from Sokol-Hessner et al (2015) were also collected over two experimental sessions on separate days. The following analyses include risky choices made both in the placebo and propranolol conditions and any group differences were examined with interaction terms (e.g. medication x past outcome) in the models reported below. Given that the previous study reported less risk-taking on the second day of the study, we accounted for day in all models in this analysis [9].

### **Risk-taking**

We accounted for effects of trial-level variables on risk-taking by regressing choice_t_ (guaranteed alternative = 0; gamble = 1) on risky gain_t_, risky loss_t_, safe_t_, and day, (where risky gain, risky loss and safe variables are in dollar amounts; Model 9). Unsurprisingly, risk-taking increased with larger risky gains (*β* = 9.1(.27), *p* = 2 x 10^-16^), and decreased with larger risky losses (*β*= 12.1(.25), *p* = 2 x 10^-16^) and safe amounts (*β* = -18.7(.54), *p* = 2 x 10^-16^). Risk-taking also decreased on the second day (*β* = -.2(.02), *p* = 2 x 10^-16^), as previously found[9].

To examine whether risk-taking was context-dependent at the immediate timescale, we regressed choice_t_ on outcome_t-1_ in addition to the trial-level variables and day (Model 10). Risk-taking decreased following large outcomes (outcome_t-1_ *β* = -.95(.11), *p* = 2 x 10^-16^; SI Fig. 2a) replicating the negative effect of past outcome in both the main text and previous work[1]. The main effects of trial-level variables and day remained (risky gain_t_ *β* = 9.15(.27), *p* = 2 x 10^-16^ ; risky loss_t_ *β*= 12.2(.25), *p* = 2 x 10^-16^ ; safe_t_ *β* = -18.8(.55), *p* = 2 x 10^-16^ ; day *β* = -.2(.02), *p* = 2 x 10^-16^)^[[1]](#footnote-1)^.

Examining the role of neighborhood effects in this dataset was complicated as the choices were not ordered into runs of similar value followed by shifts. Thus, we conducted an exploratory analysis testing for potential changes in risk-taking as a function of the difference between the mean EV on the current trial and 1) the mean EV on the previous trial, or 2) the mean EV averaged across several previous trials as an analogue for positive and negative shifts. Examining risk-taking following a change in EV from that of both a single previous trial and several previous trials in a task without intentional temporal structure could help establish whether the context-dependent behavior at the neighborhood timescale in the main text was in fact driven by runs of similar trials followed by shifts or was simply due to trial-by-trial shifts in value. This analysis should be considered strictly exploratory as this task did not have intentional temporal structure and thus was not designed to answer questions about neighborhood-level context. Moreover, averaging the mean EV over several trials is an imperfect measure, excluding other important information such as the variance across the trials (e.g. the average mean EV across three trials would be identical with mean EV values of $2/$10/$18 and $10/$10/$10, despite significant differences in similarity across trials), let alone other factors.

Because we tested the effect of positive and negative shift amount in the main text, here we included regressors for both positive and negative mean EV difference. In model 11a, the mean EV difference regressors captured the difference between the mean EV on the current trial and the average mean EV across the 3 previous trials (e.g. mean EV_t_ – average mean EV on trials t-1, t-2, and t-3). Model 11b was identical, save that it used the average mean EV from 20 previous trials. Both models (11a and 11b) included the trial-level variables, day, and outcome_t-1_. The results from model 11a revealed a significant effect of negative mean EV difference (*β* = 1.5(.4), *p* = .0003; effect size plotted in SI Fig. 2b), but not positive mean EV difference (*β* = -.56(.66), *p* = .4), with an identical pattern in model 11b (negative mean EV difference *β* = 5.9(.9), *p* = 5.7 x 10^-11^; positive mean EV difference *β* = -1.7(1.3), *p* = .19). Models 11a and 11b replicated the effects of trial-level variables, day, and outcome_t-1_ (See Supplementary Materials Table 2 for full regression results.)_._ Thus, in this exploratory analysis, there was an effect of negative mean EV difference (and not positive mean EV difference) whereas in the main text, there was an effect of positive shift (and not negative shift). However, the directions of both effects were consistent (i.e. more risk-taking when the change in value was more positive, and vice versa) suggesting that the asymmetry per se may be less robust than the overall direction of the effect.

Next, we examined whether risk-taking changed as a function of a change in mean EV from trial-to-trial. The presence of such effect would suggest that the effects of both positive shift amount in the main text and the negative mean EV difference in models 11a and 11b could be explained by a simple trial-wise change in value, rather than a truly contextual effect at the neighborhood level. We found no significant effect of a change in positive mean EV (β = -.02(.02), p = .33) or negative mean EV (β = -.008(.009), p = .37; Model 11c) in addition to the main effects of trial-level variables, day, and outcomet-1. While exploratory, these results have two potentially important general implications. First, neighborhood-level contextual effects may be present even in settings without intentional temporal structure. Second, observing that shifts in value from one trial to the next only affect risk-taking if multiple trials are taken into account suggests that participants implicitly or explicitly tracked, to some extent, the neighborhood context.

To examine whether risk-taking changed as a function of temporal context on the global level and whether there was any interaction between the global and immediate level (i.e. outcome_t-1_ x cumulative earnings_t_ as noted in the main text), we regressed choice_t_ on cumulative earnings_t_, linear expectation_t_, an interaction between cumulative earnings_t_ and outcome_t-1_, in addition to trial-level variables, day, and outcome_t-1_ (Model 12a). Note that while cumulative earnings generally increased across the task, cumulative earnings and trial are less correlated in the Sokol-Hessner et al (2015) dataset because cumulative earnings could decrease due to occasional loss outcomes (whereas in the main text, outcomes could be only either zero or positive, resulting in cumulative earnings that were strictly non-decreasing). We identified only a marginal main effect of cumulative earnings_t_ (*β* = .03(.02), *p* = .09), a significant main effect of linear expectation_t_ (*β* = -.63(.2), *p* = .0001), and a significant interaction between cumulative earnings_t_ and outcome_t-1_(*β* = .12(.04), *p* = .003; main effects of trial-level variables, day, and outcome_t-1_ remained, see Supplementary Materials Table 2 for complete regression results). These results replicate the positive interaction between cumulative earnings and previous outcomes from the main text such that participants took fewer risks following large outcomes, but only when cumulative earnings were less than or equal to linear expectations (SI Fig. 2c). We also tested whether linear expectation_t_ interacted with outcome_t-1_ to influence risk-taking (Model 12b) but found no significant interaction (*β* = -.25(.66), *p* = .7; full regression results in Supplementary Materials Table 2). The interaction between cumulative earnings_t_ and outcome_t-1_ and the lack of interaction between linear expectation_t_ and outcome_t-1_ suggests that context effects at the global timescale reflect deviations from expectations rather than changes in expectations themselves.

### **Propranolol and risk-taking**

To complement the correlational analysis of arousal and risk-taking in the main text, we tested whether the pharmacological interference with arousal by propranolol caused changes in the overall effects of temporal context. While this analysis is limited in the same way as the behavioral analysis (the lack of intentional temporal structure limits our ability to resolve contextual effects), it nevertheless can provide evidence pertinent to our overarching question about the role of arousal in contextual effects. Specifically, it provides a critical causal test for the results in the main text, which demonstrated that arousal is related to temporal context in risk-taking. If propranolol appears to change the relationship between the three timescales of temporal context and risk-taking, then that would suggest that arousal causally drives temporal context effects in risky decision-making. If propranolol has no consistent effect on temporal context and risk-taking relative to placebo, then that would instead indicate that arousal may be the result but not the cause of temporal context effects in risk-taking.

Propranolol is lipophilic, which means it acts both in the peripheral and central nervous system and has dose-dependent pharmacokinetics. This means that body size matters when administering propranolol. Because all participants in this study received the same (80mg) dose of propranolol, we adjusted for body size when examining the effect of propranolol, as was done in the original study by using the median split in BMI to characterize participants into low and high BMI groups [9].

We were interested in whether propranolol (accounting for dose-dependence by including the BMI group term) modulated the two main contextual behavioral effects on risk-taking that were observed in the main text and replicated in the secondary reanalysis – the negative effect of outcome_t-1_, and the positive interaction between outcome_t-1_ and cumulative earnings_t_. In model 13, we regressed choice_t_ on a 3-way interaction between outcome_t-1_, propranolol, and BMI group, and a 4-way interaction between outcome_t-1_, cumulative earnings_t_, propranolol, and BMI group in addition to trial-level variables, day, and all other main effects and interactions (e.g. risky gain_t,_ risky loss_t_, safe_t_, outcome_t-1_, propranolol, etc; see Supplementary Materials Table 2 for full regression results). Model 13a revealed no significant interaction between propranolol, BMI group, and outcome_t-1_ (*β* = .02(.4), *p* = .96), or between propranolol, BMI group, outcome_t-1_ and cumulative earnings_t_ (*β* = .09(.08), *p* = .29). The main effects of trial-level variables, day, outcome_t-1_, and linear expectation_t_, and the interaction between outcome_t-1_ and cumulative earnings remained.

We observed two trending interactions in model 13. The first was between cumulative earnings_t_, outcome_t-1_, and propranolol (*β* = -.15(.08), *p* = .06) such that the effect of the interaction between cumulative earnings_t_ and outcome_t-1_ on risk-taking was slightly weaker for participants on propranolol relative to the placebo. The second trending interaction was between cumulative earnings_t_, outcome_t-1_, and BMI group (*β* = -.1(.06), *p* = .08), where the effect of the interaction between cumulative earnings and outcome on risk-taking was weaker for the low BMI group relative to the high BMI group. This raises the possibility that prior research linking high BMI levels to altered risk-taking [43–45] may have unappreciated contextual dependence, though the relative weakness of these effects requires significant caution in interpretation.

Additional analyses accounting for the main effect of negative mean EV difference and a potential interaction with propranolol and BMI group did not indicate any effect of propranolol on positive or negative shifts (models 14a-b Supplementary Materials Table 2).

Given that propranolol attenuates physiological arousal responses, a strong effect of propranolol on the contextual effects observed in risk-taking behavior would suggest a causal role for arousal in underlying context-dependence in risky monetary decision-making. On the other hand, a lack of an effect of propranolol on context-dependence in risk-taking would suggest that physiological arousal does not cause the context-dependent effects we observed at the three timescales of temporal context. Our reanalysis of the propranolol data aligns with the latter rather than the former in that we find no strong effect of propranolol on the relationship between risk-taking and temporal context. However, such interpretation is complicated by the lack of intentional temporal structure in the propranolol choice set which may have reduced our ability to probe the relationship between temporal context and arousal.


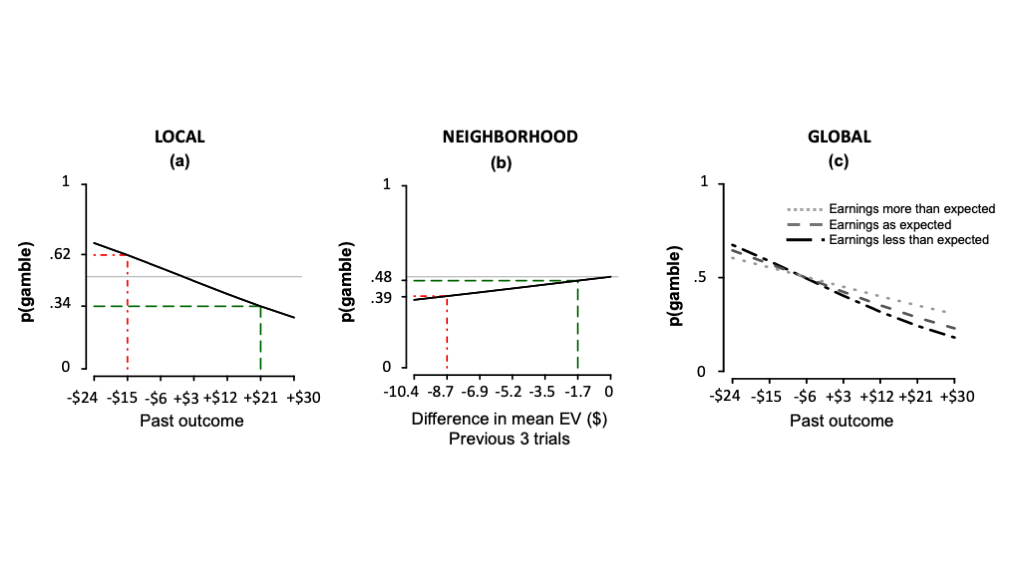


**SI Fig. 2. Visualizing the effect sizes of three temporal timescales of risky decision-making in the secondary reanalysis.** (a) Immediate timescale. Risk-taking decreases following a large outcome (green) relative to a small or negative outcome (red), assuming indifference (probability of gambling = 0.5; grey horizontal line) on the current trial. (b) Neighborhood timescale. Risk-taking increases following a large positive shift or change in mean expected value(green) relative to a trial following a small shift or negative change in mean expected value(red), assuming indifference (probability of gambling = .5; grey horizontal line) on the current trial and a past outcome of $0. (c) Global timescale interacts with immediate timescale. The effect of past outcome on risk-taking is negative when earnings are more than expected and becomes less negative (c) when cumulative earnings are more than expected (assuming indifference on the current trial).

**Ethics approval (secondary reanalysis)**

The University of Denver’s Institutional Review Board determined that this project did not require IRB review (January 18, 2019)

**Online Repositories**

Both the data and scripts (for data cleaning, preprocessing, and analysis) are located online. The data for these analyses can be accessed at <https://osf.io/a7nvx/>. The scripts used in data cleaning and analysis are located at <https://github.com/sokolhessnerlab/vic>.

**References**

1. Brooks HR, Sokol-Hessner P. Quantifying the immediate computational effects of preceding outcomes on subsequent risky choices. Sci Rep. 2020;10(1):1–10.

2. Brevers D, He Q, Xue G, Bechara A. Neural correlates of the impact of prior outcomes on subsequent monetary decision-making in frequent poker players. Biol Psychol. 2017 Mar 1;124:30–8.

3. Juechems K, Balaguer J, Ruz M, Summerfield C. Ventromedial Prefrontal Cortex Encodes a Latent Estimate of Cumulative Reward. Neuron. 2017;93(3):705–14.

4. Hytönen K, Baltussen G, van den Assem MJ, Klucharev V, Sanfey AG, Smidts A. Path dependence in risky choice: Affective and deliberative processes in brain and behavior. J Econ Behav Organ. 2014 Nov 1;107(PB):566–81.

5. Post T, Van Den Assem MJ, Baltussen G, Thaler RH. Deal or no deal? decision making under risk in a large-payoff game show. Am Econ Rev. 2008 Mar;98(1):38–71.

6. Suhonen N, Saastamoinen J. How Do Prior Gains and Losses Affect Subsequent Risk Taking? New Evidence from Individual-Level Horse Race Bets. Manag Sci. 2018;64(6):2797–808.

7. Thaler RH, Johnson EJ. Gamblng with the house money and trying to break even: The effects of prior outcomes on risky choice. Manag Sci. 1990;36(6):643–60.

8. Sokol-Hessner P, Hsu M, Curley NG, Delgado MR, Camerer CF, Phelps EA. Thinking like a trader selectively reduces individuals’ loss aversion. Proc Natl Acad Sci. 2009;106(13):5035–40.

9. Sokol-Hessner P, Lackovic SF, Tobe RH, Camerer CF, Leventhal BL, Phelps EA. Determinants of Propranolol’s Selective Effect on Loss Aversion. Psychol Sci. 2015 Jul 16;26(7):1123–30.

10. Sokol-Hessner P, Hartley CA, Hamilton JR, Phelps EA. Interoceptive ability predicts aversion to losses. Cogn Emot. 2015 May 19;29(4):695–701.

11. Sokol-Hessner P, Raio CM, Gottesman SP, Lackovic SF, Phelps EA. Acute stress does not affect risky monetary decision-making. Neurobiol Stress. 2016 Dec 1;5:19–25.

12. Pabst S, Schoofs D, Pawlikowski M, Brand M, Wolf OT. Paradoxical effects of stress and an executive task on decisions under risk. Behav Neurosci. 2013;

13. Pabst S, Brand M, Wolf OT. Stress effects on framed decisions: There are differences for gains and losses. Front Behav Neurosci. 2013;

14. Porcelli AJ, Delgado MR. Acute stress modulates risk taking in financial decision making. Psychol Sci. 2009;

15. Kluen LM, Agorastos A, Wiedemann K, Schwabe L. Cortisol boosts risky decision-making behavior in men but not in women. Psychoneuroendocrinology. 2017;

16. Solway A, Lohrenz T, Montague PR. Loss Aversion Correlates With the Propensity to Deploy Model-Based Control. Front Neurosci. 2019 Sep 6;13.

17. Ortiz-Teran E, Ortiz T, Turrero A, Lopez-Pascual J. Neural implications of investment banking experience in decision-making under risk and ambiguity. J Neurosci Psychol Econ. 2019;

18. Sokol-Hessner P, Camerer CF, Phelps EA. Emotion regulation reduces loss aversion and decreases amygdala responses to losses. Soc Cogn Affect Neurosci. 2013 Mar;8(3):341–50.

19. Gardner M, Steinberg L. Peer influence on risk taking, risk preference, and risky decision making in adolescence and adulthood: An experimental study. Dev Psychol. 2005;

20. Chumbley JR, Krajbich I, Engelmann JB, Russell E, Van Uum S, Koren G, et al. Endogenous Cortisol and Loss Aversion. Psychol Sci. 2014;25(11):2102–5.

21. Starcke K, Wolf OT, Markowitsch HJ, Brand M. Anticipatory Stress Influences Decision Making Under Explicit Risk Conditions. Behav Neurosci. 2008;

22. Imas A. The realization effect: Risk-taking after realized versus paper losses. Am Econ Rev. 2016;106(8):2086–109.

23. Sokol-Hessner P, Rutledge RB. The Psychological and Neural Basis of Loss Aversion. Curr Dir Psychol Sci. 2019 Feb 1;28(1):20–7.

24. Walasek L, Stewart N. How to Make Loss Aversion Disappear and Reverse: Tests of the Decision by Sampling Origin of Loss Aversion. J Exp Psychol Gen. 2015 Jun 1;144(1):7–11.

25. Gilaie-Dotan S, Tymula A, Cooper N, Kable JW, Glimcher PW, Levy I. Neuroanatomy predicts individual risk attitudes. J Neurosci. 2014;34(37):12394–401.

26. Sheng F, Ramakrishnan A, Seok D, Zhao WJ, Thelaus S, Cen P, et al. Decomposing loss aversion from gaze allocation and pupil dilation. Proc Natl Acad Sci U S A. 2020;117(21).

27. Rigoli F, Chew B, Dayan P, Dolan RJ. Learning contextual reward expectations for value adaptation. J Cogn Neurosci. 2017;30(1):50–69.

28. Rigoli F, Rutledge RB, Dayan P, Dolan RJ. The influence of contextual reward statistics on risk preference. NeuroImage. 2016 Mar 1;128:74–84.

29. Rigoli F, Friston KJ, Dolan RJ. Neural processes mediating contextual influences on human choice behaviour. Nat Commun. 2016 Aug 18;7.

30. Rigoli F, Mathys C, Friston KJ, Dolan RJ. A unifying Bayesian account of contextual effects in value-based choice. PLoS Comput Biol. 2016;13(10):1–28.

31. Rigoli F, Rutledge RB, Chew B, Ousdal OT, Dayan P, Dolan RJ. Dopamine increases a value-independent gambling propensity. Neuropsychopharmacology. 2016;41(11):2658–67.

32. Grether DM, Plott CR. Economic theory of choice and the preference reversal phenomenon. Am Econ Rev. 1979;69(4):623–38.

33. FeldmanHall O, Glimcher PW, Baker A, Phelps EA. Emotion and decision-making under uncertainty: Phsyiological arousal predicts increased gambling during ambiguity but not risk. J Exp Psychol Gen. 2016;145(10):1255–62.

34. Levy I, Snell J, Nelson AJ, Rustichini A, Glimcher PW. Neural representation of subjective value under risk and ambiguity. J Neurophysiol. 2010;103(2).

35. Lazzaro SC, Rutledge RB, Burghart DR, Glimcher PW. The impact of menstrual cycle phase on economic choice and rationality. PLoS ONE. 2016;11(1).

36. Xue G, Lu Z, Levin IP, Bechara A. The impact of prior risk experiences on subsequent risky decision-making: The role of the insula. NeuroImage. 2010;50(2).

37. Koop GJ, Johnson JG. The use of multiple reference points in risky decision making. J Behav Decis Mak. 2012;25(1).

38. Lee J. The effect of the background risk in a simple chance improving decision model. J Risk Uncertain. 2008;36(1).

39. O’brien RM. A Caution Regarding Rules of Thumb for Variance Inflation Factors. Qual Quant. 2007 Sep 11;41(5):673–90.

40. Dormann CF, Elith J, Bacher S, Buchmann C, Carl G, Carré G, et al. Collinearity: a review of methods to deal with it and a simulation study evaluating their performance. Ecography. 2013 Jan;36(1):27–46.

41. Brooks HR, Sokol-Hessner P. Data for Brooks & Sokol-Hessner (under revision) [Internet]. 2023. Available from: osf.io/a7nvx

42. Brooks HR, Sokol-Hessner P. GitHub Repository for Multiple timescales of temporal context in risky choice: Behavioral identification and relationships to physiological arousal [Internet]. 2023. Available from: https://github.com/sokolhessnerlab/vic

43. Ratcliff MB, Jenkins TM, Reiter-Purtill J, Noll JG, Zeller MH. Risk-taking behaviors of adolescents with extreme obesity: Normative or not? Pediatrics. 2011;127(5).

44. Navas JF, Vilar-López R, Perales JC, Steward T, Fernández-Aranda F, Verdejo-García A. Altered decision-making under risk in obesity. PLoS ONE. 2016;11(6).

45. Clifton EAD, Perry JRB, Imamura F, Lotta LA, Brage S, Forouhi NG, et al. Genome–wide association study for risk taking propensity indicates shared pathways with body mass index. Commun Biol. 2018;1(1).

1. This analysis reflects 47 study participants and 14,206 trials. Roughly 25% of these choices (a total of 3,581 trials from 24 participants; those made by participants on day 1 in the placebo condition) were examined in Brooks & Sokol-Hessner (2020). Those 3,581 trials comprised 15.3% of the data analyzed in that paper. Brooks & Sokol-Hessner (2020) analyzed the effects of previous outcomes but did not analyze the effect of propranolol as we do here, nor any temporal contexts other than ‘local’ (i.e. did not examine neighborhood or global contexts), making the analyses here largely independent both in terms of data and analytic scope. [↑](#footnote-ref-1)
