# Supplementary material for: Multiple timescales of temporal context in risky choice: Behavioral identification and relationships to physiological arousal
Source: PLoS One. 2024 Jan 19;19(1):e0296681. doi: 10.1371/journal.pone.0296681 (PMC10798524; doi:10.1371/journal.pone.0296681)
Supplement: S1 Table — (PDF) [file pone.0296681.s002.pdf]

	Number of trials	Risky gain amounts	Risky loss amounts	Guaranteed Alternative ($)	Trial type	Choice display	Decision window	ISI	Outcome display	ITI
**Main Text**	171 trials (n=8)<br>204 trials (n=5)<br>240 trials (n=49)	+$.50 to +$70	$0	+$.25 to +$35	All gain-only	2s	2s	.5s	1s	1.5s to 4.5s
**Secondary Reanalysis**	150 trials	+$2 to +$30	$0 to -$24	$0 to +$12	30/150 gain-only<br><br>120/150 gain-loss	2s	2s	1s	1s	1s to 3s

Table S1. Summary of risky decision-making task design and timing for the main text and secondary reanalysis reported in the Supplemental Materials. For full details, including the design features of gain-only and gain-loss trials as well as choice set structure for both studies, see Methods and Figure 1 in the current manuscript, the Supplemental Materials, and Sokol-Hessner et al (2015).
