# Supplementary material for: Multiple timescales of temporal context in risky choice: Behavioral identification and relationships to physiological arousal
Source: PLoS One. 2024 Jan 19;19(1):e0296681. doi: 10.1371/journal.pone.0296681 (PMC10798524; doi:10.1371/journal.pone.0296681)
Supplement: S2 Table — (PDF) [file pone.0296681.s003.pdf]

SI Table 2. Generalized linear modeling results using the "lme4" package (R version 4.0.0; “lme4” version 1.1-23).

Main Text

Model 1 = glmer(choice(t) ~ 0 + risky gain amount(t) + safe amount(t) + magnitude(t) + (0 + risky gain amount(t) + safe amount(t) | Subject ID), data, family = "binomial")

Model 1 results

AIC	BIC	logLik	deviance	df.resid
14644	14689.3	-7316	14632	13998

Fixed effects

<table>
<tr><th></th><th>Estimate</th><th>Std Error</th><th>z value</th><th>Pr(>|z|)</th></tr>
<tr><td>risky gain amount(t)</td><td>30.877</td><td>2.879</td><td>10.727</td><td>< 2e-16 ***</td></tr>
<tr><td>safe amount(t)</td><td>-13.174</td><td>5.19</td><td>-2.538</td><td>0.0111 *</td></tr>
<tr><td>magnitude(t)</td><td>-49.506</td><td>8.896</td><td>-5.565</td><td>2.62e-08 ***</td></tr>
</table>

Calculate predicted values from model 1 prior to link function:

predicted values = pred(model 1, type= "link")

Model 2 = glmer(choice(t) ~ 0 + outcome(t-1) + (1|Subject ID), data, family = "binomial", offset = predicted values)

Model 2 results

AIC	BIC	logLik	deviance	df.resid
14025.1	14040.2	-7010.6	14021.1	13940

Fixed effects

<table>
<tr><th></th><th>Estimate</th><th>Std Error</th><th>z value</th><th>Pr(>|z|)</th></tr>
<tr><td>outcome(t-1)</td><td>-0.14853</td><td>0.06124</td><td>-2.425</td><td>0.0153 *</td></tr>
</table>

Model 2a = glmer(choice(t) ~ 0 + outcome(t-1) + outcome(t-2) + outcome(t-3) + (1|Subject ID), data, family = "binomial", offset = predicted values)

Model 2a results

AIC	BIC	logLik	deviance	df.resid
13853.5	13883.7	-6922.8	13845.5	13814

Fixed effects

<table>
<tr><th></th><th>Estimate</th><th>Std Error</th><th>z value</th><th>Pr(>|z|)</th></tr>
<tr><td>outcome(t-1)</td><td>-0.30149</td><td>0.08454</td><td>-3.566</td><td>0.000362 ***</td></tr>
</table>

outcome(t-2)	0.14595	0.08498	1.717	0.085889
outcome(t-3)	0.09132	0.08464	1.079	0.28065

Model 3a = glmer(choice(t) ~ 0 + outcome(t-1) + positive shift amount(t) + negative shift amount(t) + (1|Subject ID), data, family = "binomial", offset = predicted values)

Model 3a results

AIC	BIC	logLik	deviance	df.resid
14005.4	14035.6	-6998.7	13997.4	13938

Fixed effects

	Estimate	Std Error	z value	Pr(>|z|)
outome(t-1)	-0.18331	0.06052	-3.029	0.00246 **
positive shift amount(t)	4.79699	0.99266	4.832	1.35e-06 ***
negative shift amount(t)	-0.36913	0.9001	-0.41	0.68173

Model 3b = glmer(choice(t) ~ 0 + outcome(t-1) + positive shift amount(t) + positive shift amount(t-1) + (1|Subject ID), data, family = "binomial", offset = predicted values)

Model 3b results

AIC	BIC	logLik	deviance	df.resid
14003.2	14033.4	-6997.6	13995.2	13938

Fixed effects

	Estimate	Std Error	z value	Pr(>|z|)
outcome(t-1)	-0.19347	0.06045	-3.2	0.00137 **
positive shift amount(t)	4.59259	1.00959	4.549	5.39e-06 ***
positive shift amount(t-1)	1.531	0.99774	1.534	0.12491

Model 3c = glmer(choice(t) ~ 0 + outcome(t-1) + positive shift amount(t) + positive shift amount(t)*outcome(t-1) + (1|Subject ID), data, family = "binomial", offset = predicted values)

Model 3c results

AIC	BIC	logLik	deviance	df.resid
14004	14034.1	-6998	13996	13938

Fixed effects

	Estimate	Std Error	z value	Pr(>|z|)
outcome(t-1)	-0.16994	0.06016	-2.825	0.00473 **
positive shift amount(t)	6.20723	1.41259	4.394	1.11e-05 ***
positive shift amount(t)*outcome(t-1)	-7.70914	5.40107	-1.427	0.15348

Model 4a = glmer(choice(t) ~ 0 + outcome(t-1) + positive shift amount(t) + cumulative earnings(t) + linear expectation(t) + (1ISubject ID), data, family = "binomial", offset = predicted values)

Model 4a results

AIC	BIC	logLik	deviance	df.resid
13991.3	14029	-6990.7	13981.3	13937

Fixed effects

	Estimate	Std Error	z value	Pr(>Izl)
outcome(t-1)	-0.34749	0.07617	-4.562	5.07e-06 ***
positive shift amount(t)	4.78708	0.99825	4.795	1.62e-06 ***
cumulative earnings(t)	0.75778	0.27833	2.723	0.00648 **
linear expectation(t)	-0.43454	0.21381	-2.032	0.04212 *

Model 4b = glmer(choice(t) ~ 0 + outcome(t-1) + positive shift amount(t) + cumulative earnings(t) + cumulative earnings(t)*outcome(t-1) + linear expectation(t) + (1ISubject ID), data, family = "binomial", offset = predicted values)

Model 4b results

AIC	BIC	logLik	deviance	df.resid
13974.6	14019.8	-6981.3	13962.6	13936

Fixed effects

	Estimate	Std Error	z value	Pr(>Izl)
outcome(t-1)	-0.7082	0.1131	-6.259	3.86e-10 ***
positive shift amount(t)	5.2581	1.0118	5.197	2.03e-07 ***
cumulative earnings(t)	0.378	0.2889	1.309	0.191
linear expectation(t)	-0.2582	0.2164	-1.193	0.233
cumulative earnings(t)*outcome(t-1)	1.2627	0.2921	4.323	1.54e-05 ***

Model 5a = lmer(SCR following outcome(t) ~ 1 + outcome(t) + (1ISubject ID), data)

Model 5a results

AIC	BIC	logLik	deviance	df.resid
-1883.6	-1854.6	945.8	-1891.6	10300

Fixed effects

	Estimate	Std Error	df	t value	Pr(>Itl)
intercept	0.1674	0.0094	53.3	17.7240	<2e-16 ***
outcome(t)	-0.0130	0.0093	10280.0	-1.3960	0.163

Model 5b = lmer(SCR following outcome(t) ~ 1 + positive shift amount(t) + (1|Subject ID), data)

Model 5b results

AIC	BIC	logLik	deviance	df.resid
-1883.2	-1854.3	945.6	-1891.2	10300

Fixed effects

	Estimate	Std Error	df	t value	Pr(>|t|)
intercept	0.1644	0.0091	46.12	18.095	<2e-16 ***
positive shift amount(t)	-0.1321	0.1042	10260	-1.268	0.205

Model 5c = lmer(SCR following outcome(t) ~ 1 + cumulative earnings(t) + linear expectation(t) + (1|Subject ID), data)

Model 5c results

AIC	BIC	logLik	deviance	df.resid
-2093.4	-2057.2	1051.7	-2103.4	10299

Fixed effects

	Estimate	Std Error	df	t value	Pr(>|t|)
intercept	0.1101	0.0096	63.44	11.459	< 2e-16 ***
cumulative earnings(t)	0.1621	0.0462	3315	3.51	0.000455 ***
linear expectation(t)	-0.0160	0.0355	3528	-0.451	0.652231

Model 5d = lmer(SCR following outcome(t) ~ 1 + cumulative earnings(t)*outcome(t) + (1|Subject ID), data)

Model 5d results

AIC	BIC	logLik	deviance	df.resid
-2091.8	-2048.4	1051.9	-2103.8	10298

Fixed effects

	Estimate	Std Error	df	t value	Pr(>|t|)
intercept	0.1132	0.0108	101.20	10.442	<2e-16 ***
outcome(t)	-0.0115	0.0174	10280	-0.658	0.51
cumulative earnings(t)	0.1380	0.0146	10300	9.458	<2e-16 ***
cumulative earnings(t)*outcome(t)	0.0124	0.0388	10280	0.32	0.749

Model 6a = glmer(choice(t) ~ 0 + outcome(t-1) + positive shift amount(t) + cumulative earnings(t)*outcome(t-1) + SCR following outcome(t-1) + (1ISubject ID), data, family = "binomial", offset = predicted values)

Model 6a results

AIC	BIC	logLik	deviance	df.resid
10039.4	10082.8	-5013.7	10027.4	10252

Fixed effects

	Estimate	Std Error	z value	Pr(>lzl)
outcome(t-1)	-0.7521	0.1354	-5.555	2.78e-08 ***
positive shift amount(t)	4.7522	1.20814	3.933	8.37e-05 ***
cumulative earnings(t)	0.21491	0.08972	2.395	0.01661 *
cumulative earnings(t)*outcome(t-1)	1.14658	0.35044	3.272	0.00107 **
SCR following outcome(t-1)	-0.09447	0.10792	-0.875	0.3814

Model 6b = glmer(choice(t) ~ 0 + outcome(t-1) + positive shift amount(t) + SCR following outcome(t-1)*outcome(t-1) + (1ISubject ID), data, family = "binomial", offset = predicted values)

Model 6b results

AIC	BIC	logLik	deviance	df.resid
10070.3	10106.5	-5030.2	10060.3	10253

Fixed effects

	Estimate	Std Error	z value	Pr(>lzl)
outcome(t-1)	-0.18145	0.08575	-2.116	0.034333 *
positive shift amount(t)	4.35229	1.19558	3.64	0.000272 ***
SCR following outcome(t-1)	0.09605	0.13411	0.716	0.473895
SCR following outcome(t-1)* outcome(t-1)	-0.14738	0.41899	-0.352	0.725032

Model 7 = lmer(SCR during decision phase(t) ~ 1 + choice(t) + (1ISubject ID), data)

Model 7 results

AIC	BIC	logLik	deviance	df.resid
-1575.2	-1548.5	791.6	-1583.2	5828

Fixed effects

	Estimate	Std Error	df	t value	Pr(>ltl)
intercept	0.1436	0.0135	25.68	10.621	6.87e-11 ***
choice(t)	0.0008	0.0029	5826	0.286	0.775

Model 8a = lmer(SCR during decision phase(t) ~ 1 + outcome(t-1) + positive shift amount(t) + cumulative earnings(t) + linear expectation(t) + (1|Subject ID), data)

Model 8a results

AIC	BIC	logLik	deviance	df.resid
-1632.4	-1585.7	823.2	-1646.4	5799

Fixed effects

	Estimate	Std Error	df	t value	Pr(>|t|)
intercept	0.13019	0.01458	36.94	8.928	9.32e-11 ***
outcome(t-1)	-0.02226	0.01142	5788.31	-1.949	0.05140 .
positive shift amount(t)	0.40963	0.13219	5784.59	3.099	0.00195 **
cumulative earnings(t)	-0.04378	0.05855	2603.87	-0.748	0.45467
linear expectation(t)	0.06715	0.04568	2743.29	1.47	0.14168

Model 8b = lmer(SCR during decision phase(t) ~ 1 + positive shift amount(t) + positive shift amount(t-1) + (1|Subject ID), data)

Model 8b results

AIC	BIC	logLik	deviance	df.resid
-1620.7	-1587.4	815.4	-1630.7	5801

Fixed effects

	Estimate	Std Error	df	t value	Pr(>|t|)
intercept	0.14179	0.01343	25.88	10.561	7.07e-11 ***
positive shift amount(t)	0.39837	0.13123	5779.87	3.036	0.00241 **
positive shift amount(t-1)	-0.17054	0.13123	5779.87	-1.3	0.1938

Model 8c = glmer(choice(t) ~ 0 + outcome(t-1) + positive shift amount(t) + cumulative earnings(t)*outcome(t-1) + SCR during decision phase(t) + (1|Subject ID), data, family = "binomial", offset = predicted values)

Model 8d results

AIC	BIC	logLik	deviance	df.resid
5366.2	5406.2	-2677.1	5354.2	5800

Fixed effects

	Estimate	Std Error	z value	Pr(>|z|)
outcome(t-1)	-0.77901	0.18191	-4.282	1.85e-05 ***
positive shift amount(t)	4.51789	1.64779	2.742	0.00611 **
cumulative earnings(t)	0.12934	0.12046	1.074	0.28295

cumulative earnings(t)*outcome(t-1)	1.34873	0.46039	2.93	0.00339 **
SCR during decision phase(t)	-0.04317	0.14696	-0.294	0.76892

Model 8d = lmer(SCR during decision phase(t) ~ 1 + positive shift amount(t) + choice(t) + positive shift amount(t)*choice(t) + (1|Subject ID), data)

Model 8c results

	AIC	BIC	logLik	deviance	df.resid
	-1581.5	-1541.5	796.8	-1593.5	5826

Fixed effects

	Estimate	Std Error	df	t value	Pr(>ltl)
intercept	0.1420	0.0135	25.77	10.495	8.49e-11 ***
positive shift amount(t)	0.3431	0.1336	5806	2.568	0.0102 *
choice(t)	-0.0002	0.0030	5827	-0.071	0.9437
positive shift amount(t)*choice(t)	0.1937	0.1338	5808	1.447	0.1479

Additional analyses (reported in supplemental text)

Outcomes on the 3 previous trials

glmer(choice(t) ~ 0 + outcome(t-1) + outcome(t-2) + outcome(t-3) + (1|Subject ID), data, family = "binomial", offset = predicted values)

Results

	AIC	BIC	logLik	deviance	df.resid
	13853.5	13883.7	-6922.8	13845.5	13814

Fixed effects

	Estimate	Std Error	z value	Pr(>lzl)
outcome(t-1)	-0.30149	0.08454	-3.566	0.000362 ***
outcome(t-2)	0.14595	0.08498	1.717	0.085889 .
outcome(t-3)	0.09132	0.08464	1.079	0.28065

Positive shift (step-wise regression, accounting for past outcome effect first, then using predicted values and the offset function in R)

glm(choice(t) ~ 0 + positive shift (t), data, family, offset = predicted values)

Results

AIC	deviance	df.resid
13996	14017	13994

Fixed effects

	Estimate	Std Error	z value	Pr(>lzl)
positive shift(t)	4.7197	0.9892	0.771	1.83e-06 ***

Outcomes x Positive Shift

glmer(choice(t) ~ 0 + outcome(t-1) + positive shift(t) + outcome(t-1) x positive shift(t) + (1lSubject ID), data, family = "binomial", offset = predicted values)

Results

AIC	BIC	logLik	deviance	df.resid
14004	14034.1	-6998	13996	13938

Fixed effects

	Estimate	Std Error	z value	Pr(>lzl)
outcome(t-1)	-0.16994	0.06016	-2.825	0.00473 **
positive shift(t)	6.20737	1.43286	4.332	1.48e-05 ***
outcome(t-1) x positive shift (t)	-7.70941	5.46331	-1.411	0.15821

Trial-level model with expected value difference
(mean EV of gamble - mean EV of safe option)

glmer(choice(t) ~ 0 + EV difference(t) + (0 + EV difference(t) l Subject ID), data, family = "binomial")

Results

AIC	BIC	logLik	deviance	df.resid
16460.2	16475.3	-8228.1	16456.2	14002

Fixed effects

	Estimate	Std Error	z value	Pr(>lzl)
EV difference(t)	32.036	2.913	11	< 2e-16 ***

Calculate predicted values from model above prior to link function:

predicted values = pred(model, type= "link")

3 timescales model using predicted values from EV difference model above (this model is identical to model 4a reported in the main text)

glmer(choice(t) ~ 0 + outcome(t-1) + positive shift amount(t) + cumulative earnings(t) + linear expectation(t) + (1|Subject ID), data, family = "binomial", offset = predicted values)

Results

	AIC	BIC	logLik	deviance	df.resid
	14312.3	14350	-7151.2	14302.3	13937

Fixed effects

	Estimate	Std Error	z value	Pr(>|z|)
outcome (t-1)	-0.41936	0.08562	-4.898	9.68e-07 ***
positive shift amount (t)	4.37113	0.993	4.402	1.07e-05 ***
cumulative earnings (t)	1.3873	0.43446	3.193	0.00141 **
linear expectations (t)	-0.77435	0.33341	-2.323	0.02021 *

Secondary Reanalysis (reported in supplemental text)

Model 9 = glmer(choice(t) ~ 1 + risky gain amount(t) + risky loss amount(t) + safe amount(t) + day + (1|Subject ID), data, family = "binomial")

Model 9 results

	AIC	BIC	logLik	deviance	df.resid
	13550.8	13596.1	-6769.4	13538.8	14020

Fixed effects

	Estimate	Std Error	z value	Pr(>|z|)
intercept	0.13705	0.13746	0.997	0.319
risky gain amount(t)	9.05427	0.26737	33.865	<2e-16 ***
risky loss amount(t)	12.14485	0.24667	49.234	<2e-16 ***
safe amount(t)	-18.6547	0.53944	-34.581	<2e-16 ***
day	-0.1883	0.02132	-8.834	<2e-16 ***

Model 10 = glmer(choice(t) ~ 1 + risky gain amount(t) + risky loss amount(t) + safe amount(t) + day + outcome(t-1) + (1|Subject ID), data, family = "binomial")

Model 10 results

	AIC	BIC	logLik	deviance	df.resid
	13385.3	13438.1	-6685.6	13371.3	13925

	Fixed effects			
	Estimate	Std Error	z value	Pr(>lzl)
intercept	0.18369	0.13848	1.326	0.185
risky gain amount(t)	9.14857	0.27044	33.828	<2e-16 ***
risky loss amount(t)	12.21458	0.24902	49.051	<2e-16 ***
safe amount(t)	-18.81036	0.54616	-34.441	<2e-16 ***
day	-0.19025	0.02147	-8.863	<2e-16 ***
outcome(t-1)	-0.95299	0.1118	-8.524	<2e-16 ***

Model 11a = glmer(choice(t) ~ 1 + risky gain amount(t) + risky loss amount(t) + safe amount(t) + day + outcome(t-1) + positive mean EV difference(3 previous trials) + negative mean EV difference(3 previous trials) + (1lSubject ID), data, family = "binomial")

Model 11a results

AIC	BIC	logLik	deviance	df.resid
13180	13247.8	-6581	13162	13735

	Fixed effects			
	Estimate	Std Error	z value	Pr(>lzl)
intercept	0.21926	0.13977	1.569	0.11672
risky gain amount(t)	9.13973	0.28742	31.8	< 2e-16 ***
risky loss amount(t)	12.03993	0.26381	45.638	< 2e-16 ***
safe amount(t)	-18.50302	0.66597	-27.784	< 2e-16 ***
day	-0.18946	0.02164	-8.754	< 2e-16 ***
outcome(t-1)	-0.90435	0.11906	-7.596	3.05e-14 ***
positive mean EV difference (3 previous trials)	-0.55934	0.65874	-0.849	0.39582
negative mean EV difference (3 previous trials)	1.49395	0.40901	3.653	0.00026 ***

Model 11b = glmer(choice(t) ~ 1 + risky gain amount(t) + risky loss amount(t) + safe amount(t) + day + outcome(t-1) + positive mean EV difference(20 previous trials) + negative mean EV difference(20 previous trials) + (1lSubject ID), data, family = "binomial")

Model 11b results

AIC	BIC	logLik	deviance	df.resid
11664	11730.6	-5823	11646	12137

	Fixed effects			
	Estimate	Std Error	z value	Pr(>lzl)
intercept	0.26505	0.14426	1.837	0.0662 .
risky gain amount(t)	8.54531	0.35695	23.94	< 2e-16 ***

risky loss amount(t)	10.94793	0.32259	33.938	< 2e-16 ***
safe amount(t)	-16.3237	1.01378	-16.102	< 2e-16 ***
day	-0.17706	0.02301	-7.695	1.42e-14 ***
outcome(t-1)	-0.94941	0.12179	-7.795	6.43e-15 ***
positive mean EV difference (20 previous trials)	-1.7192	1.29925	-1.323	0.1858
negative mean EV difference (20 previous trials)	5.91915	0.90364	6.55	5.74e-11 ***

Model 11c = glmer(choice(t) ~ 1 + risky gain amount(t) + risky loss amount(t) + safe amount(t) + day + outcome(t-1) + positive mean EV difference(t-1) + negative mean EV difference(t-1) + (1lSubject ID), data, family = "binomial")

Model 11b results

AIC	BIC	logLik	deviance	df.resid
13387.3	13455.2	-6684.7	13369.3	13923

Fixed effects

	Estimate	Std Error	z value	Pr(>lzl)
intercept	0.18241	0.13897	1.313	0.189
risky gain amount(t)	9.24361	0.28223	32.752	< 2e-16 ***
risky loss amount(t)	12.30474	0.25851	47.599	< 2e-16 ***
safe amount(t)	-18.55055	0.61185	-30.319	< 2e-16 ***
day	-0.19021	0.02147	-8.86	< 2e-16 ***
outcome(t-1)	-1.05837	0.13594	-7.786	6.93e-15 ***
positive mean EV difference(t-1)	-0.47944	0.49303	-0.972	0.331
negative mean EV difference(t-1)	-0.23689	0.26382	-0.898	0.369

Model 12a = glmer(choice(t) ~ 1 + risky gain amount(t) + risky loss amount(t) + safe amount(t) + day + outcome(t-1) + cumulative earnings(t) + cumulative earnings(t)*outcome(t-1) + linear expectation(t) + (1lSubject ID), data, family = "binomial")

Model 12a results

AIC	BIC	logLik	deviance	df.resid
13359	13434.5	-6669.5	13339	13922

Fixed effects

	Estimate	Std Error	z value	Pr(>lzl)
intercept	0.38796	0.14514	2.673	0.007519 **
risky gain amount(t)	9.15671	0.27107	33.78	< 2e-16 ***

risky loss amount(t)	12.30689	0.25084	49.062	< 2e-16 ***
safe amount (t)	-18.77966	0.54702	-34.331	< 2e-16 ***
day	-0.1903	0.0215	-8.852	< 2e-16 ***
outcome(t-1)	-1.4255	0.19633	-7.261	3.85e-13 ***
cumulative earnings(t)	0.03045	0.0178	1.711	0.087082 .
cumulative earnings(t)*outcome(t-1)	0.12023	0.04116	2.921	0.003485 **
linea expectation(t)	-0.62624	0.16214	-3.862	0.000112 ***

Model 12b = glmer(choice(t) ~ 1 + risky gain amount(t) + risky loss amount(t) + safe amount(t) + day + outcome(t-1) + cumulative earnings(t) + cumulative earnings(t)*outcome(t-1) + linear expectation(t) + linear expectation(t)*outcome(t-1) + (1ISubject ID), data, family = "binomial")

Model 12b results

AIC	BIC	logLik	deviance	df.resid
13360.9	13443.9	-6669.4	13338.9	13921

Fixed effects

	Estimate	Std Error	z value	Pr(>lzl)
intercept	0.38386	0.14553	2.638	0.008349 **
risky gain amount(t)	9.15997	0.27127	33.767	< 2e-16 ***
risky loss amount(t)	12.30689	0.25091	49.05	< 2e-16 ***
safe amount(t)	-18.7809	0.54713	-34.326	< 2e-16 ***
day	-0.19029	0.0215	-8.852	< 2e-16 ***
outcome(t-1)	-1.37926	0.23106	-5.969	2.38e-09 ***
cumulative earnings(t)	0.0291	0.01816	1.602	0.109069
cumulative earnings(t)*outcome(t-1)	0.1404	0.06731	2.086	0.036985 *
linear expectation(t)	-0.60966	0.16798	-3.629	0.000284 ***
linear expectation(t)*outcome	-0.2497	0.65936	-0.379	0.704911

Model 13 = glmer(choice(t) ~ 1 + risky gain amount(t) + risky loss amount(t) + safe amount(t) + day + outcome(t-1) + linear expectation(t) + outcome(t-1)*propranolol*BMI group + cumulative earnings(t)*outcome(t-1)*propranolol*BMI group + (1ISubject ID), data, family = "binomial")

Model 13a results

AIC	BIC	logLik	deviance	df.resid
13341.1	13507	-6648.5	13297.1	13910

Fixed effects

	Estimate	Std Error	z value	Pr(>lzl)
intercept	0.364023	0.15051	2.419	0.015582 *
risky gain amount(t)	9.19292	0.27203	33.794	< 2e-16 ***
risky loss amount(t)	12.36572	0.2521	49.05	< 2e-16 ***

safe amount(t)	-18.86449	0.54933	-34.341	< 2e-16 ***
day	-0.212417	0.02198	-9.662	< 2e-16 ***
outcome(t-1)	-1.589866	0.27421	-5.798	6.71e-09 ***
cumulative earnings(t)	0.029982	0.01971	1.521	0.128163
propranolol	0.063047	0.07875	0.801	0.423377
BMI group	0.015244	0.14246	0.107	0.914781
linear expectation(t)	-0.696657	0.16347	-4.262	2.03e-05 ***
outcome(t-1)*cumulative earnings(t)	0.209629	0.05855	3.581	0.000343 ***
outcome(t-1)*propranolol	0.257081	0.39404	0.652	0.51413
outcome(t-1)*BMI group	0.11994	0.27329	0.439	0.660753
cumulative earnings(t) *propranolol	0.018938	0.01693	1.119	0.263295
cumulative earnings(t)*BMI group	-0.001266	0.01192	-0.106	0.915415
propranolol*BMI group	0.095575	0.07896	1.21	0.226097
outcome(t-1)*cumulative earnings(t) *propranolol	-0.153465	0.08294	-1.85	0.064264 .
outcome(t-1)*cumulative earnings(t) *BMI group	-0.101838	0.05843	-1.743	0.081352 .
outcome(t-1)*propranolol *BMI group	0.018403	0.39407	0.047	0.962752
cumulative earnings(t)* propranolol*BMI group	0.020518	0.01695	1.21	0.226095
outcome(t-1)*cumulative earnings(t)*propranolol*BMI group	0.087981	0.08294	1.061	0.288797

Model 14a = glmer(choice(t) ~ 1 + risky gain amount(t) + risky loss amount(t) + safe amount(t) + day + linear expectation(t) + cumulative earnings(t)*outcome(t-1) + positive mean EV difference(3 previous trials)*propranolol*BMI group + negative mean EVdifference(3 previous trials)*propranolol*BMI group + (1|Subject ID), data, family = "binomial")

Model 13b results

	AIC	BIC	logLik	deviance	df.resid
	13141.6	13299.7	-6549.8	13099.6	13723

Fixed effects

	Estimate	Std Error	z value	Pr(>|z|)
intercept	0.31979	0.14993	2.133	0.032932 *
risky gain amount(t)	9.17266	0.28799	31.85	< 2e-16 ***
risky loss amount(t)	12.16227	0.26614	45.699	< 2e-16 ***
safe amount(t)	-18.62511	0.66612	-27.96	< 2e-16 ***

day	-0.20772	0.02209	-9.405	< 2e-16 ***
outcome(t-1)	-1.45792	0.20026	-7.28	3.34e-13 ***
cumulative earnings(t)	0.04394	0.01808	2.431	0.015068 *
linear expectation(t)	-0.66121	0.1648	-4.012	6.01e-05 ***
positive mean EV difference (3 previous trials)	-0.343	0.69602	-0.493	0.622153
negative mean EV difference (3 previous trials)	1.13443	0.5733	1.979	0.047843 *
propranolol	0.14613	0.06262	2.334	0.019621 *
BMI group	0.03603	0.13898	0.259	0.795458
outcome(t-1)*cumulative earnings(t)	0.14121	0.04145	3.406	0.000659 ***
positive mean EV difference (3 previous trials)*BMI group	-0.48185	0.32673	-1.475	0.140275
positive mean EV difference (3 previous trials)*propranolol	-0.24482	0.46229	-0.53	0.59641
BMI group*propranolol	0.15744	0.06332	2.487	0.012900 *
negative mean EV difference (3 previous trials)*BMI group	0.47714	0.56055	0.851	0.394662
negative mean EV difference (3 previous trials)*propranolol	0.79192	0.79372	0.998	0.318415
positive mean EV difference (3 previous trials)*propranolol *BMI group	-0.0665	0.46242	-0.144	0.885653
negative mean EV difference (3 previous trials)*propranolol *BMI group	-1.12779	0.79403	-1.42	0.155509

Model 14b = glmer(choice(t) ~ 1 + risky gain amount(t) + risky loss amount(t) + safe amount(t) + day + linear expectation(t) cumulative earnings(t)*outcome(t-1) + positive mean EV difference(20 previous trials)*propranolol*BMI group + negative mean EV difference(20 previous trials)*propranolol*BMI group + (1ISubject ID), data, family = "binomial")

Model 13c results

	AIC	BIC	logLik	deviance	df.resid

	11631.1	11786.6	-5794.6	11589.1	12125
	Fixed effects				
	Estimate	Std Error	z value	Pr(>lzl)	
intercept	0.34547	0.1597	2.163	0.030515 *	
risky gain amount(t)	8.54938	0.36129	23.663	< 2e-16 ***	
risky loss amount(t)	11.02754	0.33151	33.264	< 2e-16 ***	
safe amount(t)	-16.60003	1.02157	-16.25	< 2e-16 ***	
day	-0.19428	0.02348	-8.273	< 2e-16 ***	
outcome(t-1)	-1.56783	0.23664	-6.625	3.47e-11 ***	
cumulative earnings(t)	0.0678	0.01911	3.548	0.000389 ***	
linear expectation(t)	-0.77842	0.1796	-4.334	1.46e-05 ***	
positive mean EV difference(20 previous trials)	-1.22627	1.34166	-0.914	0.360722	
negative mean EV difference(20 previous trials)	5.19937	1.21197	4.29	1.79e-05 ***	
propranolol	0.17063	0.0771	2.213	0.026899 *	
BMI group	-0.02482	0.14353	-0.173	0.86273	
outcome(t-1)*cumulative earnings(t)	0.13911	0.04614	3.015	0.002568 **	
positive mean EV difference (20 previous trials)*BMI	-0.1521	0.35312	-0.431	0.666668	
positive mean EV difference (20 previous	-0.47288	0.49992	-0.946	0.344188	
BMI group*propranolol	0.16468	0.07785	2.115	0.034395 *	
negative mean EV difference (20 previous	-1.11613	1.07983	-1.034	0.301315	
negative mean EV difference (20 previous	2.03217	1.533	1.326	0.184966	
positive mean EV difference (20 previous trials)* propranolol*BMI group	-0.07082	0.49993	-0.142	0.887348	
negative mean EV diffrence (20 previous trials)* propranolol*BMI group	-1.20454	1.53297	-0.786	0.43201	
