# Supplementary material for: Multiple timescales of temporal context in risky choice: Behavioral identification and relationships to physiological arousal
Source: PLoS One. 2024 Jan 19;19(1):e0296681. doi: 10.1371/journal.pone.0296681 (PMC10798524; doi:10.1371/journal.pone.0296681)
Supplement: Attachment — Submitted filename: Response to Reviewers.docx [file pone.0296681.s004.docx]

Response to Reviewers

PONE-D-23-21525

Title: Multiple timescales of temporal context in risky choice: Behavioral identification and relationships to physiological arousal

Hayley R. Brooks & Peter Sokol-Hessner

*Thank you for the opportunity to revise our manuscript! We were excited that the editor and reviewers shared our perspective that this study and its findings have intriguing and important implications for the science of decision-making, and deeply appreciated your thorough, constructive, and engaged assessment of our research. We have made many changes to our paper as a result of your thoughtful reviews and believe that it has been markedly improved by your suggestions, corrections, and questions.*

*Below, we quote the original feedback from the Editor and all reviewers and respond to each question or concern in turn. Our responses are in italic blue text for ease of processing. While we discuss in depth nearly every change below, as a summary, we have made significant new additions to the literature discussed in the paper which more broadly and effectively connects our findings to others’ research, we have performed a number of additional analyses to address potential statistical concerns, and we have included the full analytic code for all regressions, among numerous other, smaller changes.*

*Thank you again for the opportunity to revise our manuscript, and for your shared excitement for this research.*

*Dr. Brooks and Dr. Sokol-Hessner*

Editor Comments:

The research conducted by Brooks and Sokol-Hessner presents intriguing experiments that demonstrate the independent influences of reward history at multiple time scales on risk-taking behavior. The manuscript is generally well-crafted; however, the models posited for underlying mechanisms lack specificity due to the absence of detailed computational models. While this ambiguity leaves room for robust discussion concerning potential mechanisms, the authors have commendably summarized relevant, particularly recent, literature. I have two primary suggestions for additional scholarly works that could enrich the manuscript's discussion section.

Firstly, the work of Kolling et al. provides precedent for the modulation of risk-taking decisions based on contextual variables. Specifically, they investigated the influence of time pressure on the attainment of a specified level of cumulative reward. I recommend that the authors consider comparing their findings with those of Kolling et al. to more comprehensively address the contextual effects on risk-taking decisions.

Reference:
Kolling, N., Wittmann, M., & Rushworth, M. F. (2014). Multiple neural mechanisms of decision making and their competition under changing risk pressure. Neuron, 81(5), 1190-1202.

Secondly, the relationship between arousal and contextual shifts has previously been explored by Joshua Gold and Matt Nassar. They assessed the impact of sudden environmental changes on arousal systems and subsequent behavioral adjustments. A comparison between these findings and the current study could provide valuable insights.

Reference:
Nassar, M., Rumsey, K., Wilson, R. et al. Rational regulation of learning dynamics by pupil-linked arousal systems. Nat Neurosci 15, 1040–1046 (2012).

*Thank you for pointing us to these two very relevant and interesting pieces of literature. We have now added both papers to the discussion (Nassar et al (2012) middle of page 35, lines 746-750 and Kolling et al (2014) at the beginning of page 38, lines 801-803).*

Minor Point:
To enhance clarity concerning the regression analyses, I concur with Reviewer 2 in suggesting that the authors delineate all the equations related to the regression models, either in the Methods or Results section. Providing tables containing estimated coefficients, statistical significance, and criteria for fitting performance (e.g., AIC, BIC, LOOC) would be beneficial for the reader.

*We accidentally submitted our original supplemental file without including two tables (describing task design and regression equations and output). We sincerely apologize for this oversight and thank you for bringing this to our attention. We have now included both the comprehensive list of regression results in the supplemental file (S3 Table 2) and the relevant regression equations (methods section) in the main text.*

Reviewers' comments:

Reviewer's Responses to Questions
**Comments to the Author**

1. Is the manuscript technically sound, and do the data support the conclusions?

The manuscript must describe a technically sound piece of scientific research with data that supports the conclusions. Experiments must have been conducted rigorously, with appropriate controls, replication, and sample sizes. The conclusions must be drawn appropriately based on the data presented.
Reviewer #1: Yes
Reviewer #2: Partly

2. Has the statistical analysis been performed appropriately and rigorously?
Reviewer #1: Yes
Reviewer #2: Yes

3. Have the authors made all data underlying the findings in their manuscript fully available?

The [PLOS Data policy](https://urldefense.com/v3/__http:/www.plosone.org/static/policies.action*sharing__;Iw!!NCZxaNi9jForCP_SxBKJCA!UCpiXVlHuyoZ1hKJduowjSfIlOeXbTzm_0GUIXFd6TzV-0Lf55CvWXm-SYqkMXXZTg10VFkHL-MjVnA4l5F-TiYk$) requires authors to make all data underlying the findings described in their manuscript fully available without restriction, with rare exception (please refer to the Data Availability Statement in the manuscript PDF file). The data should be provided as part of the manuscript or its supporting information, or deposited to a public repository. For example, in addition to summary statistics, the data points behind means, medians and variance measures should be available. If there are restrictions on publicly sharing data—e.g. participant privacy or use of data from a third party—those must be specified.
Reviewer #1: Yes
Reviewer #2: Yes

4. Is the manuscript presented in an intelligible fashion and written in standard English?

PLOS ONE does not copyedit accepted manuscripts, so the language in submitted articles must be clear, correct, and unambiguous. Any typographical or grammatical errors should be corrected at revision, so please note any specific errors here.
Reviewer #1: Yes
Reviewer #2: Yes

5. Review Comments to the Author

Please use the space provided to explain your answers to the questions above. You may also include additional comments for the author, including concerns about dual publication, research ethics, or publication ethics. (Please upload your review as an attachment if it exceeds 20,000 characters)

Reviewer #1: This paper presents an analysis of temporal context in risky decision making, including a measure of physiolgoical arousal (skin conductance response). The key findings are that large positive outcomes increased risk-taking at short and intermediate timescales, and decreased risk-taking at long timescales (but only when cumulative earnings were sufficiently low relative to expectations). The relationship between skin conductance and risk-taking at different timescales showed several effects but lacked a clean correspondence with the behavioral findings.

Overall, I thought this was a very comprehensively analyzed dataset. The paper is clearly written and well organized. I think these findings will be very valuable to any researcher working in this area. I didn't see any major issues in the manuscript. Of course, the theoretical intepretation of these results, given the somewhat inconsisent nature of the skin conductance data, remains to be seen, but I think this kind of paper serves as a good challenge for theorists.

*We are very appreciative of the reviewer’s thoughtful read of this paper as a meaningful contribution to the field’s understanding of risky decision-making and psychophysiology. We agree that these findings have implications for a range of research questions into contextual effects in human behavior. We touch on many of these implications in the discussion in hopes that others will find these connections useful and insightful. We have addressed the reviewer’s minor comments in the manuscript. Thank you for your time spent reviewing our paper!*

Minor:

p. 10: "built in into" → "built into"
*Thank you for catching this! We have made this change.*

p. 27: "may have it roots" → "may have its roots"

*We have made this correction as well.*

Reviewer #2: This manuscript by Roper and Sokol-Hessner examines how risky decision-making in human subjects depends on contextual factors, specifically past outcomes at immediate, short-term, and long-term timescales. Using a standard risk decision-task with fluctuating temporal structure (primarily runs of trials at given EVs, switching in unsignaled blocks), the authors show via regression analyses that risk taking depended on context at different timescales: immediate (past trial outcome), local neighborhood (post-run shift effects), and global (cumulative potential earnings compared to expectation). Skin conductance responses were related to the local and global timescales, but were not directly related to changes in risky choice behavior. This is an interesting set of findings that relate to a growing literature on contextual effects in economic choice behavior. The authors provide a nice extension to some of their previous work by examining temporal context effects at different timescales. The paper is on the while relatively clearly written, but the authors should clarify some issues of analysis and interpretation.

*We are very appreciative to the reviewer for volunteering their time and expertise to provide thoughtful feedback on our manuscript. We are gratified that they agree with us that these findings are well-situated in the current, growing literature on context in economic choice behavior. We have carefully considered and addressed the reviewer’s points below with additional analyses, discussions, and revisions to the manuscript.*

Major points
(1) Multiple timescales versus a time-varying influence

One of the most interesting aspects of the results is the finding of behavioral temporal context effects at different time scales. However, the authors could do a better job justifying their particular choice of analyses to examine short/medium/long timescales. For example, the influence of past outcomes at varying timescales have been examined in many different scenarios as a weighted (e.g. exponential kernel) sum of past trial rewards (Khaw et al for reward adaptation in behavior, Sugrue et al for matching law neural coding, etc.). Did the authors try a similar approach to determine the effect of past trials (e.g. a lagged regression)?

*We agree with the reviewer that there are many possible analysis choices. Our decision to examine previous outcome amount on the previous trial only was determined by results in our previously published paper (Brooks & Sokol-Hessner, 2020, Scientific Reports) where we did not find any meaningful effects of previous outcome amount on risk-taking that extended beyond the most recent trial (t-1). We replicated this pattern in the current dataset and briefly listed the results below and have now included the full model results in the supplemental file on pages 2-3 and in S3 Table 2.*

| Choice ~ 0 + Outcome_t-1_+ Outcome_t-2_+ Outcome_t-3_ + (1 \|subject)  Note: Like the models in the paper, we included the offset specification when using R’s GLMER function. | | | | |
| --- | --- | --- | --- | --- |
| Fixed effects: |  |  |  |  |
|  | Estimate | Std. Error | z-value | p-value |
| Outcome_t-1_ | -0.30149 | 0.08454 | -3.566 | 0.000362 *** |
| Outcome_t-2_ | 0.14595 | 0.08498 | 1.717 | 0.085889 . |
| Outcome_t-3_ | 0.09132 | 0.08464 | 1.079 | 0.280650 |

*Most broadly, we certainly agree with the reviewer’s overarching point that there may well be other additional forms of temporal context effects than those described here, but argue that we describe at least 3 novel levels of temporal context in risky decision-making, with the acknowledgment that there may be others in tasks that have different structure or timescales than that used here.*

The neighborhood effect the authors find is interesting, but it’s not clear to me that it’s strictly a local/medium timescale effect. The authors show a change in risk-taking following a shift in EV between runs, but only on the first trial following the shift (and only for positive shifts). This seems to argue against a strict intermediate-timescale effect, which should persist for trials (as an intermediate-defined context - like running average - changes incrementally with additional trials after the shift). Rather than a temporal effect, this seems like an effect more consistent with a violation of expectation or a novelty effect (something the authors touch on in the Discussion). Additionally, can the authors discuss why they thing the effect is evident for positive but not negative shifts?

*We agree that it can be challenging to make sense of the contextual effect at the neighborhood timescale. The effect of shift on risk-taking extends only to the trial immediately following a positive shift and is only present when the recent history of values prior to a shift has been relatively consistent. We do not see the positive shift effect in datasets where the choice set lacks intentional temporal structure (i.e. where the values of risky gain and safe values are random and not grouped into runs of trials with a similar expected value). Because the value history is necessary to create the sense of novelty and subsequently observe the positive shift effect on risk-taking, we think of this effect as temporal. Put another way, the antecedents of the effect are on a neighborhood level, even if the behavioral consequence is much briefer.*

*The presence of the positive vs negative shift effect on risk-taking is of course less powerful and robust relative to other effects we’ve observed (e.g., previous outcome amount). We think that positive shifts are more likely to induce the sense of novelty because they result in novel/infrequent values (e.g., $60), whereas negative shifts lead to values that have likely been encountered previously in the task. Because we agree that readers may have a similar reaction, we now discuss this possibility in the discussion at the beginning of page 32 starting on line 670.*

(2) Clarity about regression analysis.

The paper largely relies on regression analyses to quantify context effects, and there are a few clarifications the authors could provide.

I am concerned there may be an issue with multicollinearity in Model 1 between risky gain, safe gain and magnitude. Since the task structure changes all three variables together across different trials runs (all change together with run-specific EV level), across all trials these variables may significantly covary - making parameter estimation uncertain. Any parameter estimate problems here would affect subsequent analyses that fix these regression weights while fitting others. Did the authors test for multicollinearity (e.g. by examining VIFs) in this initial regression?

*We appreciate the reviewer’s concern about collinearity. As the reviewer points out, the task structure introduces collinearity as the risky gain, safe, and magnitude all vary together across runs. Collinearity can of course lead to issues interpreting parameter estimates for a given regressor that is collinear with others. We note here that the parameter estimates in question (risky gain, safe, and magnitude) both show expected effects (e.g., effects for which we have very strong priors like observing more risk-taking as risky gain amounts rise), and perhaps most importantly, are not the central focus of our analysis. Our goal is to account for these factors, and then assess the additional impact of contextual variables. We also note that the literature on how to respond to collinearity is equivocal as to whether any of a variety of possible corrective approaches for e.g., a high VIF, are actually helpful, or if they just introduce additional issues (O’Brien, 2007; Dormann et al, 2012). We interpret this literature to persuasively argue that when collinearity occurs, it is critical to explicitly acknowledge and discuss it as a limitation, but that there is ultimately no agreed-upon way to eliminate collinearity without introducing other issues.*

*To quantitatively approach the question of whether this collinearity significantly impacted the temporal context effects that we reported in the results, we ran a similar analysis using the conservative two-step approach described in the main text. First, we fit a linear mixed effects model with choice regressed on expected value difference (i.e. mean expected value of the risky gamble – mean expected value of the safe option) to simplify the model and eliminate collinearity. Model results shown below demonstrate that risk-taking increased as the expected value difference increased (β = 32.04 (2.913), p < 2x10^-1^). We then used R’s offset function to hold constant the parameter estimates of the expected value difference in a subsequent model that tested whether risk-taking changed as a function of the three timescales of temporal context (this is the conservative two-step analytical approach we take in the manuscript). We regressed choice on past outcome amount_t-1_, positive shift amount_t_, and earnings_t_ relative to linear expectations_t_._._ We replicated all of the effects reported in the main text: risk-taking increased as past outcome decreased (β = -.42 (.09), p = 9.7x10^-7^), positive shift increased (β = 4.37 (.99), p = 1.07x10^-5^), and when earnings were more than expectations (earnings β = 1.39 (.43), p =.001, expectations β = -.77 (.33), p =.02). The full regression results are below. These results are qualitatively identical to those originally reported in the manuscript suggesting stable parameter estimates for contextual variables despite collinearity in the current-trial parameters. We now include this analysis and discussion in S1 Text on pages 4-5 in section “Testing an alternative trial-level model: Expected value difference”.*

Trial-level model with expected value difference:

choice ~ 0 + EV difference + (0 + EV difference |subject)

AIC	BIC	logLik deviance df.resid

16460.2 16475.3 -8228.1 16456.2	14002

Scaled residuals:

Min	1Q Median	3Q	Max

-5.0383 -0.9157 -0.2942 0.8106 8.3962

Random effects:

Groups	Name	Variance	Std.Dev.

subject	EV difference	578.9	24.06

Number of obs: 14004, groups: subject	, 62

Fixed effects:

Estimate	Std. Error z value	Pr(>|z|)

EV difference	32.036	2.913	11	<2e-16 ***

Temporal context model with three timescales:

Choice ~ 0 + outcome_t-1_ + positive shift_t_ + earnings_t_ + linear expectations_t_ + (1 |subject)

AIC	BIC	logLik deviance df.resid

14312.3 14350.0 -7151.2 14302.3	13937

Scaled residuals:

Min	1Q Median	3Q	Max

-5.0108 -0.6522 -0.2202 0.6864 7.6290

Random effects:

Groups	Name	Variance	Std.Dev.

subject	(Intercept)	0.8933	0.9452

Number of obs: 13942, groups: subjectIndex, 62

Fixed effects:

Estimate	Std. Error z value	Pr(>|z|)

outcome_t-1_	-0.41936	0.08562 -4.898	9.68e-07 ***

positive shift_t_	4.37113	0.99300	4.402	1.07e-05 ***

earnings_t_	1.38730	0.43446	3.193	0.00141 **

linear expectations_t_	-0.77435	0.33341 -2.323	0.02021 *

---

Signif. codes: 0 ‘***’ 0.001 ‘**’ 0.01 ‘*’ 0.05 ‘.’ 0.1 ‘ ’ 1

Correlation of Fixed Effects:

outcome_t-1_	positive shift_t_	earnings_t_

positive shift_t_	0.061

earnings_t_	-0.066	0.095

linear expectations_t_	0.066	-0.069	-0.979

For the previous trial analyses, it seems that outcome at t-1 is potentially confound with win versus loss; I believed this was examined in the authors’ previous paper but not examined here - at least please clarify that this was the assumption, and justified by previous findings.

*Thank you for pointing this out. We did indeed examine this in our previous paper and describe and justify the assumption of this analysis choice in the text:*

*“One recent study using a nearly identical task structure (8) found that risk-taking was significantly predicted by recent outcomes. The effect was driven by the immediate prior outcome amount above and beyond other factors such as the prior choice, mean expected value of prior choice options, or the prior outcome type (i.e. risky win, risky loss, or safe outcome). The effect of the previous outcome amount was also relatively short-lasting, best characterized as the effect of the immediately-preceding trial only. On the basis of these previous findings we therefore added outcome_t-1_ to the regression on choice_t_,…” (page 15)*

Additionally, I am concerned that the previous trial outcome effects (Model 2) and the neighborhood shift effects (e.g. Model 3a) reflect the same phenomenon. Model 2 shows that low past trial t-1 outcome increases risk taking. Model 3a shows that positive shifts increase risk taking, using a regressor that corresponds to the difference in EV between current trial and past trial. This regressor on shift trials covaries with the EV of the previous trial, which is correlated with the previous trial outcome. I realize the previous trial outcome is included as a covariate, but it would help if the authors examined the collinearity between shift and outcome regressors.

*We appreciate the reviewer’s concern about the potential confound between previous outcomes and positive shift amounts. If we understand correctly, the concern is that because of the task design, the range of past outcome amounts associated with large positive shifts is smaller than the range of past outcomes associated with small positive shifts. In other words, the issue could be that the increase in risk-taking following a positive shift is not due to the change in value between runs but is accounted for by the negative effect of past outcome (relatively more risk-taking following small outcomes versus large ones). In this case, following a large positive shift the previous outcome is constrained to be relatively small and therefore leads to relatively more risk-taking.*

*While this is an accurate statement of some of the structural aspects of our task, and appreciate the potential for concern, we don’t think this is an observed issue in this task and dataset for several reasons.*

*First, we note that the positive shift effect is observed in regressions which also account for the previous trial effect. If our understanding of the reviewer’s concern is accurate, then the previous trial regressor should out-perform the positive shift regressor as it would more accurately indicate the precise value that was affecting decisions (the past outcome). Nevertheless, both regressors remain significant in the same regression, suggesting that the positive shift regressor captures additional variance that the past outcome regressor does not.*

*Second, to additionally test the potential concern, we used step-wise regression. First, as in our existing analyses, we regressed out current-trial variables (like risky gain and safe), which we then held constant in a second regression on previous outcome alone. This procedure is that taken in Models 1 and 2 in the main text, described as our conservative two-step approach. We then followed the same procedure a third time to hold the estimates of Model 1 and 2 constant while regressing choice on positive shift amount in a third, new model. This approach gave all the shared variance to the previous outcome variable in Model 2. If the effect of positive shift is entirely accounted for by the past outcome effect, then we would not expect to see a significant effect of shift on risk-taking in this third, new model. However, we replicate the positive shift effect, indicating that increases in risk-taking following large positive shifts are distinct from and in addition to the effect of previous outcomes (Regression results are in the table below and we now include this in S3 Table 2). These findings are most consistent with a perspective in which the past outcome regressor and the positive shift regressor are not sharing variance around one common effect (the past outcome effect), and are instead two distinct effects.*

*Third, we also tested for an interaction between past outcome and positive shift amount and found only the main effects for outcome and positive shift as reported in the main text (outcome_t-1_ β = -0.17 (.06), p = .005; positive shift_t_ = 6.21(1.43), p = 1.48x10^-5^; outcome_t-1_ x positive shift_t_ β = -7.71(5.46), p =.16). We report the full regression results in S3 Table 2 and include these results and discussion in the S1 Text pages 3-4.*

*Fourth and finally, we note that our design protects against the confound raised by the reviewer as we have a relatively wide range of possible outcomes even when large shifts occur. The figure below plots the positive shift amount against the previous outcomes for all participants, and shows that following large shifts, the range of possible previous outcomes is roughly 60% of the possible range of outcomes throughout the task. Were this range significantly more restricted (i.e. to ≤ 10% of the range), we agree that the concern raised would be much more serious, but the very variability in the possible outcomes received at a given level reduces the intensity of this confound.*

*Together, we feel that the above argues that the shift effect is separate from the past outcome effect on risk-taking and that the design of our tasks protects against the worst version of this confound.*

Step 1: Past outcome model (same as Model 2 reported in manuscript):

Choice ~ 0 + outcome_t-1_ + (1 |subject)

AIC	BIC	logLik deviance df.resid

14025.1 14040.2 -7010.6 14021.1	13940

Scaled residuals:

Min	1Q Median	3Q	Max

-6.1625 -0.6549 -0.1561 0.6957 9.0667

Random effects:

Groups	Name	Variance Std.Dev.

subjectIndex (Intercept) 0.0009235 0.03039

Number of obs: 13942, groups: subjectIndex, 62

Fixed effects:

Estimate Std. Error z value Pr(>|z|)

pastOC1sc -0.14853	0.06124 -2.425	0.0153 *

---

Signif. codes: 0 ‘***’ 0.001 ‘**’ 0.01 ‘*’ 0.05 ‘.’ 0.1 ‘ ’ 1

Step 2:

Choice ~ 0 + positive shift_t_ (now including predicted values from past outcome model above)

Coefficients:

Estimate Std. Error z value Pr(>|z|)

shiftDiffscPOS	4.7197	0.9892	4.771 1.83e-06 ***

---

Signif. codes: 0 ‘***’ 0.001 ‘**’ 0.01 ‘*’ 0.05 ‘.’ 0.1 ‘ ’ 1

Null deviance: 14017 on 13942 degrees of freedom

Residual deviance: 13994 on 13941 degrees of freedom

(62 observations deleted due to missingness)

AIC: 13996

(3) Global context analysis.

The authors could provide a little more explanation about what the global context analyses are meant to capture exactly. The results are framed as the overall effect of a difference between cumulative earnings experienced and a linear expectation, and at first glance the relevance of these constructs to subjects is unclear. If the subjects are explicitly told that they would be rewarded for a single trial, why would they track the cumulative earnings (rather than, for example, the average)? It’s also unclear why subjects would have a linear, constantly increasing expectation; testing the piecewise linear expectation was a good addition, but still has the issue of an increasing expectation not matching the single rewarded trial structure.

In addition, it seems that both these quantities - and in particular, the linear expectation - would be confounded by a simple measure of time in the experimental session. Is there a similar effect if trial number is included in the regression?

*We can certainly understand the reviewer’s questions about the presence of global context effects. Indeed, any contextual effect on risk-taking is puzzling when individuals are rewarded for a single trial. In this way, our entire paper challenges that assumption, at multiple levels. With respect to the global timescale more specifically, we have now added additional text in both the results (Page 21, line 435) and discussion (Pages 33-34, lines 697-715; see below for text snippet) to clarify what the global context analyses are meant to capture. We feel that these revisions have improved the clarity about the global context analyses and believe the reviewer will agree.*

*“The relative weight of these two independent variables can capture when individuals are ahead or behind their expectations throughout the task.” (Results, Page 21, line 435)*

*“In our study, cumulative earnings were normatively irrelevant. They did not explicitly influence outcomes or the final payment in the current task, but their effects were nonetheless observed, suggesting that the dominant frameworks for risky decision-making may have overlooked fundamental aspects of value computation and decision-making over time. Recent research has demonstrated that individuals implicitly track cumulative earnings (11), a finding which we extend to settings in which doing so appears irrelevant, and possibly disadvantageous, to payoff (an observation that additionally applies to all context effects observed in the current study). We assumed that cumulative earnings were compared to expectations via a straightforward linear additive mechanism. While decision-makers are known to dynamically track current resources compared to a threshold or expectation in other domains of decision-making (e.g. foraging(79), which we discuss more below), the motivations for and precise functional form of such tracking in risky monetary decision-making is unclear. It is additionally possible that the effect of earnings relative to expectations is nonlinear. In particular, some evidence (12–14) suggests that any departure of earnings from expectations might lead to more risk-taking. Fitting such a form in future research will require advanced nonlinear approaches not used here as well as larger datasets with more trials and explicit measures of expectations and earnings that are dissociated from time. What is clear is that the study of risky monetary decision-making has overlooked a critical component of choice behavior.” (Discussion, Page 33-34, lines 697-715)*

Minor points

(1) It would greatly help clarity if the authors provided equations for their different regression models (preferably in the Methods, though SI would be OK). I think grouping them together in one place would help the reader see the differences and similarities between the different model versions.

*We appreciate and agree with the reviewer that providing the model equations and results would significantly improve the readability of this paper. In our initial submission, we accidently left out the two tables in the supplement, one of which included the full model equations and results for both the main analysis and the supplement. We have now included those tables in the supplement as well as included the model equations throughout the results section. Our apologies for the oversight!*

(2) Small clarification: were the L/R screen position of safe/risky gambles counterbalanced or randomized across trials?

*The location of the risky gamble and safe options on the screen were randomized across trials. We have now clarified this in the Methods section Page 9 line 169.*

(3) line 49: not clear what “risk-taking against disincentives” means; perhaps “despite incentives” might be clearer?

*We have now revised this phrase to be more clear (page 3 line 48-50):*

*“That contextual effects persist in risk-taking despite appearing disadvantageous suggests that risky decision-making may be fundamentally contextually sensitive.”*
